# A lysosome‐targeted ultra‐sensitive viscosity probe for monitoring viscosity alterations during chemotherapy

**DOI:** 10.1002/smo2.70070

**Published:** 2026-06-10

**Authors:** Haoyang Song, Hui Bian, Lei Liu, Gahyeon Park, Bingqing Sun, Qiongzheng Hu, Juyoung Yoon

**Affiliations:** ^1^ Department of Chemistry and Nanoscience Ewha Womans University Seoul South Korea; ^2^ Graduate Program in Innovative Biomaterials Convergence Ewha Womans University Seoul South Korea; ^3^ New and Renewable Energy Research Center Ewha Womans University Seoul South Korea; ^4^ College of Chemistry and Materials Engineering Anhui Science and Technology University Bengbu China; ^5^ School of Pharmaceutical Sciences Qilu University of Technology (Shandong Academy of Sciences) Jinan China; ^6^ Shandong Analysis and Test Center Qilu University of Technology (Shandong Academy of Sciences) Jinan China

**Keywords:** apoptosis, chemotherapy drug screen, ferroptosis, fluorescence imaging, lysosomal viscosity

## Abstract

Lysosomal viscosity is a key biomarker of cancer progression and chemotherapy response, but real‐time, precise monitoring remains challenging. To address this challenge, we develop a novel chemosensing platform based on a molecular rotor architecture for specific, dynamic detection of lysosomal viscosity. The platform operates via an “off‐on” switching mechanism: in low‐viscosity environments, rapid rotor rotation through the twisted intramolecular charge transfer effect quenches fluorescence; in high‐viscosity conditions, restricted rotation triggers strong emission, enabling an ultra‐sensitive and selective response. Using systematic molecular engineering and screening within this platform, the probe PMA‐H is identified as the optimal candidate, demonstrating a remarkable 187‐fold fluorescence enhancement in response to viscosity (from 0.54 to 1410 cP), excellent environmental stability with minimal interference from pH, polarity, or biomolecules, and precise lysosomal targeting. Subsequently, PMA‐H is employed to track lysosomes in HeLa cells, and it reveals alterations in lysosomal viscosity, morphology, and abundance during apoptosis, ferroptosis, cuproptosis, and zinc‐induced cell death. In general, this platform allows real‐time tracking of lysosomal viscosity fluctuations induced by various chemotherapeutic agents, highlighting its significant potential as a powerful tool for early cancer diagnostics and fundamental lysosomal research.

## Graphical abstract

A novel chemosensing platform based on a molecular rotor architecture is established for specific, dynamic detection of lysosomal viscosity. The platform allows real‐time tracking of lysosomal viscosity fluctuations induced by various chemotherapeutic agents, highlighting its significant potential as a powerful tool for early cancer diagnostics and fundamental lysosomal research.

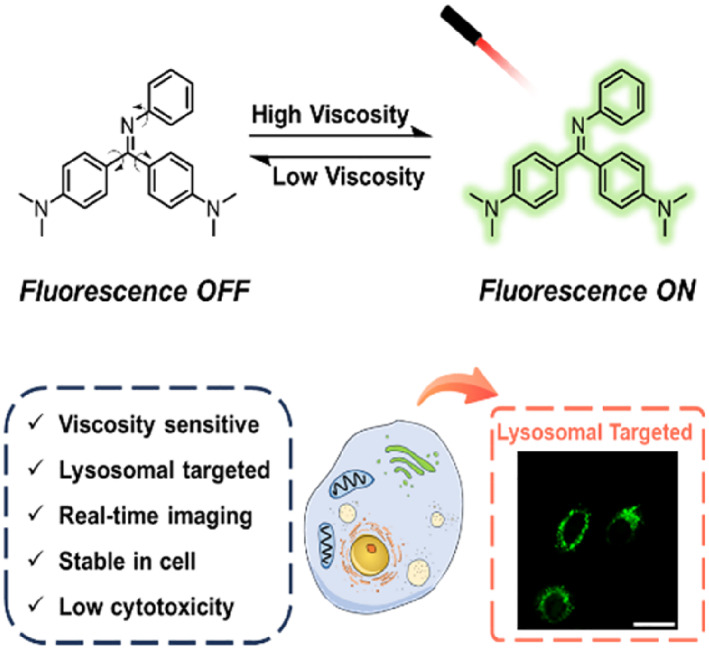

## 1 INTRODUCTION

As the primary center for degradation and metabolic regulation, lysosomes play an irreplaceable role in maintaining cellular homeostasis, regulating inflammatory responses, and modulating tumor occurrence and development.[^1^, ^2^, ^3^] An increasing number of studies have shown that lysosomal structures, functions, and behaviors are significantly affected during programmed cell death induced by anticancer drugs.[^4^, ^5^] Because chemotherapy causes a range of lysosomal dysfunctions, detection of lysosomal parameters such as viscosity, pH, polarity, and reactive oxygen species has become a research focus in studies of cancer occurrence, development, treatment, and prognosis.[^6^, ^7^] Collectively, these studies highlight the importance of detecting lysosome‐associated parameters in this field.

Of note, viscosity is one of the key physical parameters in the cellular microenvironment, directly affecting molecular diffusion, enzymatic reaction kinetics, and intracellular signal transduction.[^8^, ^9^, ^10^] Growing evidence has shown that abnormal viscosity fluctuations in organelles, especially lysosomes, are closely related to a variety of diseases, such as atherosclerosis, diabetes, inflammatory disorders, and Alzheimer's disease.[^11^, ^12^, ^13^, ^14^, ^15^] Meanwhile, as one of the important indicators of lysosomal functional state, lysosomal viscosity changes significantly during tumor occurrence and drug intervention.[^16^, ^17^] For example, rapid tumor growth induces metabolic dysregulation and excessive protein synthesis, which increase lysosomal crowding and viscosity. Consequently, elevated viscosity represents a potential biomarker for tumor diagnosis.^[18]^ In contrast, some antitumor drugs in chemotherapy can directly or indirectly alter lysosomal membrane permeabilization (LMP) or disrupt lysosomal functions, resulting in unusual accumulation of undigested macromolecules and proteases within lysosomes or translocation of them into the cytoplasm, which leads to significant changes in lysosomal viscosity.^[19]^ This process is closely related to apoptosis and inflammatory responses and directly affects the efficacy of chemotherapy drugs and the drug resistance of tumor cells.^[20]^ Therefore, monitoring changes in lysosomal viscosity is regarded as an important tool that reflects the pathological state of lesion tissue and aids early‐stage disease diagnosis, particularly in the evaluation of the therapeutic efficacy of anticancer drugs.

Fluorescence metrology offers a number of advantages for the real‐time tracking, detection, and reporting of viscosity.[^21^, ^22^, ^23^, ^24^, ^25^, ^26^, ^27^] Fluorescence‐based approaches represent the most valuable tools for intracellular viscosity sensing owing to their high spatial resolution, real‐time capability, and noninvasive features.[^28^, ^29^] The core principle in the design of viscosity‐responsive probes lies in the incorporation of freely rotating rotors in fluorophores.[^30^, ^31^, ^32^] Under low‐viscosity conditions, these rotors enable rapid dissipation of excited‐state energy through enhanced non‐radiative decay, resulting in dim fluorescence. In contrast, high‐viscosity environments restrict intramolecular rotation, thereby suppressing non‐radiative decay and restoring fluorescence emission. Consequently, changes in viscosity can be precisely identified by monitoring variations in fluorescence intensity or fluorescence lifetime. For instance, Meng and co‐workers developed a lysosome‐targeted fluorescent probe whose fluorescence intensity and lifetime both exhibited an excellent linear relationship with the viscosity.^[33]^ In addition, Zeng et al. developed a TICT‐active thioxanthene indolium dye with a 122‐fold increase in fluorescence intensity from water to 95% glycerol for monitoring viscosity changes in lysosomes.^[34]^ Although these design strategies achieve viscosity detection to a certain extent, several inherent limitations remain unavoidable. First, viscosity‐responsive probes that rely on fluorescence lifetime typically exhibit a relatively narrow range of lifetime changes, which consequently restrict the detection window. Second, most reported probes are based on intramolecular charge transfer structures, making them susceptible to responsiveness to both polarity and viscosity, thereby introducing potential interference. Third, for many organic fluorescence probes, the highly hydrophobic structures can either facilitate their insertion into protein cavities or lead to aggregation‐caused quenching or aggregation‐induced emission (AIE), which results in false‐negative or false‐positive signals. Finally, the background fluorescence of probes in low‐viscosity biological environments is a critical factor that directly affects detection accuracy. Therefore, the development of a novel probe that can respond selectively to viscosity changes, resist interference from other environmental factors, and enable background‐free detection is both highly important and urgently needed.

Herein, we developed a lysosome‐targeted background‐free chemo‐sensing platform for real‐time monitoring of subcellular viscosity changes. Three rationally designed fluorescent probes (PMA‐X) were constructed based on a molecular rotor scaffold in which donor and acceptor units are connected through an enamine bridge. This design leverages the twisted intramolecular charge transfer (TICT) mechanism. In low‐viscosity environments, free rotation of the enamine bond promotes non‐radiative decay and quenches fluorescence. In viscous media, restricted rotation suppresses the TICT process and produces strong fluorescence turn‐on. After a systematic screening, PMA‐H was identified as the optimal candidate, exhibiting the highest fluorescence quantum yield (Φ = 0.169) and a remarkable 187‐fold fluorescence enhancement in glycerol. Upon cellular uptake, PMA‐H selectively accumulated in lysosomes with excellent colocalization and reliably responded to viscosity changes within this organelle. Its utility was further demonstrated by imaging drug‐treated cancer cells, where it accurately reported pathological viscosity alterations. Compared with existing viscosity probes (Table S1), PMA‐H offers a broad detection range (0.54–1410 cP), real‐time capability, and high spatial resolution at the subcellular level, providing a robust and versatile tool for studying lysosomal function in disease contexts.

## 2 RESULTS AND DISCUSSION

### 2.1 Design and characteristics of PMA‐X

The PMA‐X probes incorporate three molecular rotors, one benzene and two *p*‐dimethylaminobenzenes connected by an enamine bridge. In this system, the two *p*‐dimethylaminobenzenes act as electron donors and transfer electrons to acceptor—enamine. The triple molecular rotors not only enhance the radiative decay of excited‐state energy in sterically restricted environments but also maintain a twisted, less‐conjugated ground‐state conformation, thereby attenuating intramolecular charge transfer in PMA‐X. Introduction of multiple rotors and modulation of spatial conformation are expected to quench fluorescence as much as possible and provide the basis for a sharp viscosity‐dependent fluorescence increase. Meanwhile, halogen substituents were designed to enable precise regulation of photoluminescence properties by modulating the electronic distribution, intramolecular charge‐transfer process, and conformational relaxation. The synthetic routes of PMA‐X are illustrated in Figure S1. The structures of all compounds were confirmed by ^1^H NMR (nuclear magnetic resonance), ^13^C NMR, and electrospray ionization high‐resolution mass spectrometry, and the corresponding spectra are provided in Figures S14–S24.

### 2.2 Photophysical properties

**FIGURE 1:**
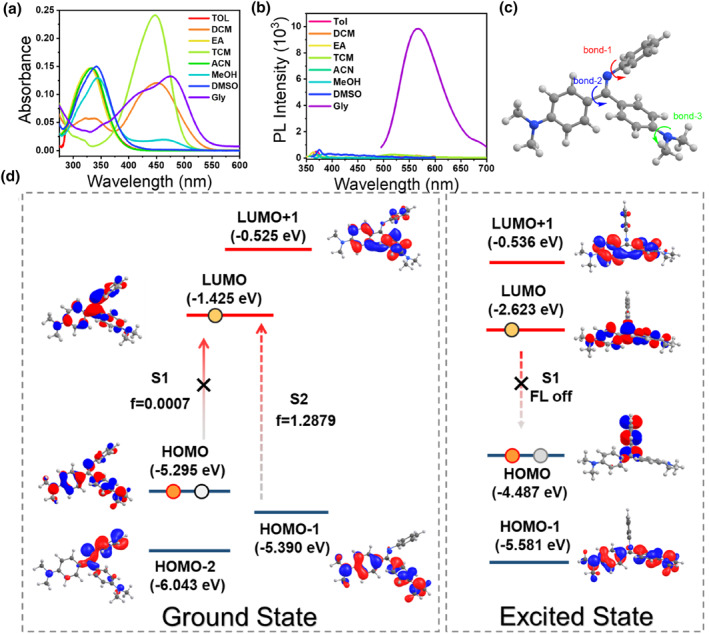
(a) Absorbance and (b) fluorescence emission spectra of PMA‐H (5 μM) in various organic solvents, including toluene, dichloromethane, ethyl acetate, trichloromethane, acetonitrile, methanol, dimethyl sulfoxide and glycerol. (c) Optimized conformation of PMA‐H in the ground state. (d) TD‐density functional theory calculation of the frontier molecular orbitals of PMA‐H in S_0_ and S_1_ states.

Next, we characterized the photophysical properties of PMA‐X. As shown in Figure 1, PMA‐X exhibited similar spectral characteristics. In the low‐polarity solvent toluene, the main absorption bands of PMA‐X were located at approximately 320 nm. With increasing solvent polarity, the absorption spectra showed a pronounced red shift. Pronounced red shifts for PMA‐H were observed in dichloromethane (peak at 447 nm), trichloromethane (peak at 450 nm), and glycerol (peak at 475 nm) (Figure 1a). Specifically, in glycerol, the maximum absorption of PMA‐H was at 475 nm with a shoulder peak at approximately 420 nm. Similar absorption profile was observed for PMA‐Br and PMA‐I, with absorption maxima at 485 nm and a shoulder peak at approximately 435 nm (Figure S2a,c). Although solvent polarity influences the absorption spectra of these molecules, our measurements reveal that most of them showed no fluorescence except in glycerol (Figures 1b and S2b,d). In glycerol, all three probes exhibited a maximum fluorescence emission peak at 567 nm, and PMA‐H showed the highest intensity.

To gain deeper insight into the intrinsic properties of the molecule, density functional theory calculations were performed using the Gaussian 16 program. As shown in Figure 1c, the optimized molecular conformation of PMA‐H in methanol exhibited a pronounced twisted conformation in which the three rotors do not coplanar. This structural feature explained the extremely low background fluorescence of the molecules in different solvents. Analysis of the molecular orbitals revealed that in both the ground and excited states, the electron densities of the HOMO (highest occupied molecular orbital) and LUMO (lowest unoccupied molecular orbital) were spatially separated, indicating significant intramolecular charge separation. Notably, in the excited state, the HOMO and LUMO were completely localized on two different moieties, signifying complete charge separation and consequent fluorescence quenching (Figures 1d, S3  and S4). Compared with the ground‐state structure, the dihedral angle defined by the four atoms forming bond 1 and bond 2 showed the largest change in angle degree, from 57° to 90°. At a dihedral angle of 90°, PMA‐H reached a stable conformation with a very low oscillator strength (0.0007), indicating negligible fluorescence emission (Table S2). A relaxed excited‐state potential energy scan of rotation around bond 1 revealed that the torsional potential energy and fluorescence intensity increased as the dihedral angle decreased, indicating that the non‐emissive excited‐state conformation is the most stable (Figure S5). Therefore, it was reasonable to infer that, in a high‐viscosity environment, the excited‐state structure cannot undergo such extensive twisting—specifically, the dihedral angle cannot reach 90°—and consequently, fluorescence emission from a more planar conformation was strong. These results suggested that PMA‐H responds to viscosity via the TICT process. Moreover, the large Stokes shift of PMA‐H (92 nm) further corroborates this conclusion by indicating conformational rearrangement (Figure S6). Meanwhile, PMA‐H showed the highest fluorescence quantum yield (Φ = 0.169), supporting that viscosity‐induced conformational changes stabilize the excited state and improve the photoluminescence efficiency.

**FIGURE 2:**
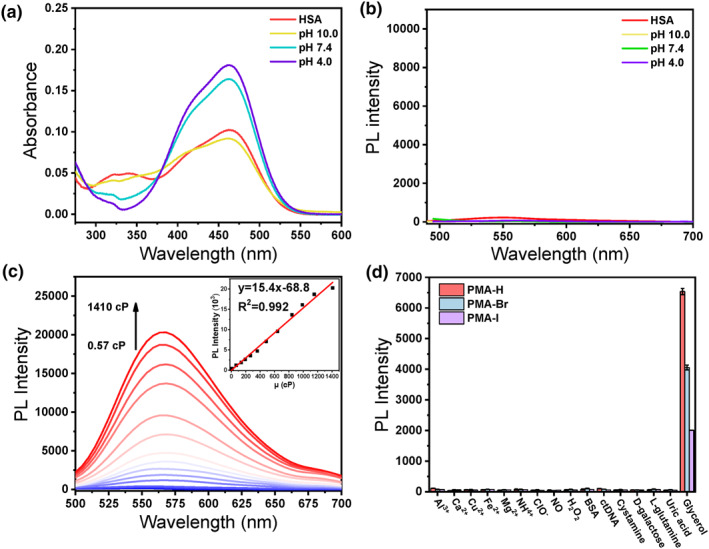
(a) Absorbance and (b) fluorescence spectra of PMA‐H (5 μM) in PBS buffer containing 5 mg/mL HSA and in PBS buffer at pH 10.0, 7.4, and 4.0. (c) Fluorescence intensity of PMA‐H (5 μM) in methanol/glycerol mixtures as viscosity increased. The inset shows the linear relationship between the dynamic viscosity of the solvents and the fluorescence intensity of PMA‐H at 565 nm (5 μM). (d) Selectivity indicated by the fluorescence responses of PMA‐H (red), PMA‐Br (blue), and PMA‐I (purple). Fluorescence responses of PMA‐H (5 μM) to different metal ions, biofunctional compounds, and some metabolic molecules (1 mM Al^3+^, 5 mM Ca^2+^, 5 mM Cu^2+^, 5 mM Fe^2+^, 5 mM Mg^2+^, 5 mM NH_4_^+^, 5 mM ClO^−^, 10 μM NO, 100 μM H_2_O_2_, 0.5 mg/mL bovine serum albumin, 20 μg/mL ctDNA, 100 μM Cystamine, 100 μM D‐Galactose, 100 μM L‐glutamine, 100 μM uric acid, and pure glycerol solvent). Data were expressed as mean ± standard error (*n* = 3). HSA, human serum albumin; PBS, phosphate‐buffered saline.

To account for aqueous biological environments, we evaluated the photophysical properties of PMA‐X in aqueous solutions (Figures 2 and S7). Owing to the strong polarity of water, the main absorption bands of PMA‐H were located around 450 nm (Figure 2a). Variations in pH did not affect the absorption peak positions, although alkaline conditions led to a slight decrease in absorption intensity; however, such conditions were rarely encountered in biological systems, particularly within lysosomes. As expected, no fluorescence emission was observed under acidic and alkaline conditions, which perfectly meets the requirement for background‐free detection (Figure 2b). More importantly, the introduction of proteins did not interfere with the photophysical properties of the molecules. After the addition of human serum albumin, a slight decrease in absorption intensity was observed, while fluorescence emission remained undetectable, indicating that the molecules are insensitive to protein interference and can effectively avoid the drawbacks commonly associated with hydrophobic fluorescent probes. In addition, the AIE performance was also examined. As shown in Figure S8, no fluorescence emission was observed under any tested conditions, demonstrating that the molecules did not exhibit AIE characteristics and therefore will not introduce false‐positive or false‐negative errors in subsequent detection and analysis.

Subsequently, the relationship between fluorescence intensity and viscosity was investigated (Figure 2c). To match the chemical environment as closely as possible, methanol and glycerol, which have similar dielectric constants but different viscosities, were mixed in different proportions as the solvent.^[35]^ As the viscosity increased, the fluorescence of PMA‐H at 567 nm gradually intensified, and the fluorescence intensity increased approximately 187‐fold. Meanwhile, the fluorescence intensity showed a good linear relationship with viscosity (*R* ^2^ = 0.992) over the viscosity range from 0.54 to 1410 cP, indicating that PMA‐H is a highly sensitive viscosity‐responsive probe. Although PMA‐Br and PMA‐I showed 114‐fold and 86‐fold increases in maximum fluorescence emission as viscosity increased (Figure S9), PMA‐H exhibited higher fluorescence brightness and better viscosity‐responsive performance, indicating that the non‐halogenated structure provided the optimal photophysical properties. Notably, PMA‐H showed no luminescence response to other small molecules widely present within cells, such as reactive oxygen species, reactive nitrogen species, amino acids, sugars, and metal ions (Figure 2d). Therefore, PMA‐H demonstrated a highly sensitive and specific response to viscosity and was nearly unaffected by other factors, which make it potential for viscosity detection under complicate biological conditions. Finally, to demonstrate the photostability of PMA‐H, the absorption and fluorescence emission spectra of PMA‐H were measured the under 460 nm laser irradiation for different durations. As seen in Figure S10, under 5 mW/cm^2^ irradiation, the decreases of relative absorbance and emission intensity were less than 10%.

### 2.3 Cytotoxicity and optimization of imaging conditions

Motivated by the sensitive response of PMA‐H to viscosity, its fluorescence imaging performance was further investigated to monitor viscosity variations at the subcellular level. Given the biocompatibility requirements for a fluorescence imaging probe, the cytotoxicity of PMA‐X was evaluated first. As shown in Figure S11, the cell viability remained above 80% when HeLa cells were exposed to 5 μM PMA‐H, whereas the cell viability treated with PMA‐Br and PMA‐I obviously decreased. The IC_50_ value of PMA‐H was approximately 6.56 mM, suggesting good biocompatibility. Subsequently, confocal laser scanning microscopy was employed to investigate the cellular uptake process. As shown in Figure S12, the fluorescence signal gradually increased with prolonged incubation time; however, the fluorescence histograms from 0.5 to 12 h showed no significant differences, indicating that the probe was rapidly internalized by cells. After 12 h of incubation, the cell morphology did not change markedly, providing additional evidence consistent with the cytotoxicity results.

### 2.4 Fluorescence imaging and colocalization

**FIGURE 3:**
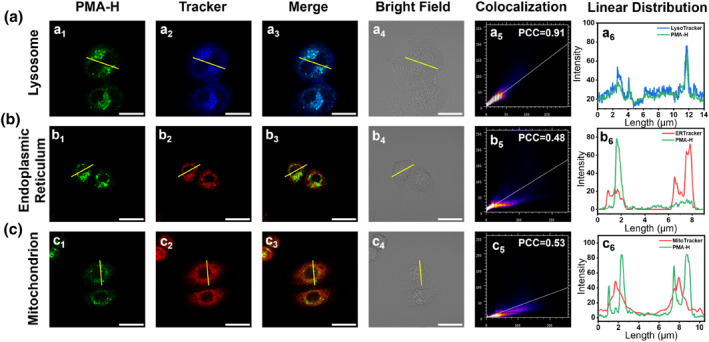
Colocalization of PMA‐H and commercial organelle dyes in HeLa cells. HeLa cells were treated with PMA‐H (5 μM) and LysoTracker™ Blue DND‐22 (a), ERTracker™ Red (b), and MitoTracker Deep Red (c) at a concentration of 10 nM. (a_1_–c_1_) Emission from the green channel (PMA‐H), *λ*_ex_ = 488 nm, collected at 500–550 nm. (a_2_) Emission from the blue channel (LysoTracker), *λ*_ex_ = 405 nm, collected at 437–477 nm. (b_2_) Emission from the red channel (ERTracker), *λ*_ex_ = 561 nm, collected at 570–620 nm. (c_2_) Emission from the deep red channel (MitoTracker), *λ*_ex_ = 638 nm, collected above 650 nm. (a_5_–c_5_) Scatter plots for colocalization analysis between commercial dyes and PMA‐H; Pearson's correlation coefficient was calculated using ImageJ2. (a_6_) LysoTracker and PMA‐H fluorescence intensity distribution maps in cells (yellow lines in a_1_–a_4_). (b_6_) ERTracker and PMA‐H fluorescence intensity distribution maps in cells (yellow lines in b_1_–b_4_). (c_6_) MitoTracker and PMA‐H fluorescence intensity distribution maps in cells (yellow lines in c_1_–c_4_). Scale bar: 20 μm.

To determine the intracellular origin of the fluorescence, HeLa cells were stained with PMA‐H (5 μM) and three commercial dyes for subcellular organelles. As shown in Figure 3a1, PMA‐H fluorescence appeared and concentrated in the form of green puncta around the nucleus. The merged image showed substantial overlap between the blue and green channels (Figure 3a3). This colocalization was supported by the intensity scatter plot and its Pearson correlation coefficient (PCC) of 0.91 (Figure 3a5), as well as by similar fluorescence intensity profiles (Figure 3a6). This evidence demonstrated strong colocalizations between PMA‐H and LysoTracker‐stained regions, indicating precise lysosomal targeting by PMA‐H. In contrast, colocalization with MitoTracker and ERTracker showed pronounced differences with minimal overlap between PMA‐H and the commercial dyes. Consistently, PCC values were low (0.48 for endoplasmic reticulum colocalization and 0.53 for Mito colocalization), and the intensity profiles differed (Figure 3b6,c6). These results support the conclusion that PMA‐H can specifically target lysosomes in living cells, enabling subcellular viscosity‐responsive fluorescence imaging.

### 2.5 Fluorescence imaging of lysosomes during chemotherapy

Given the high viscosity responsiveness, rapid cellular uptake, and lysosomal targeting capability of PMA‐H, we investigated drug‐induced changes in lysosomal viscosity to provide insights for drug screening. As representative models, lysosomal viscosity variations were examined during apoptosis, cuproptosis, and ferroptosis. Specifically, HeLa cells were incubated with different drugs for 24 h to elicit distinct pharmacological effects and then stained with 5 μM PMA‐H for 30 min to monitor lysosomal behaviors.

**FIGURE 4:**
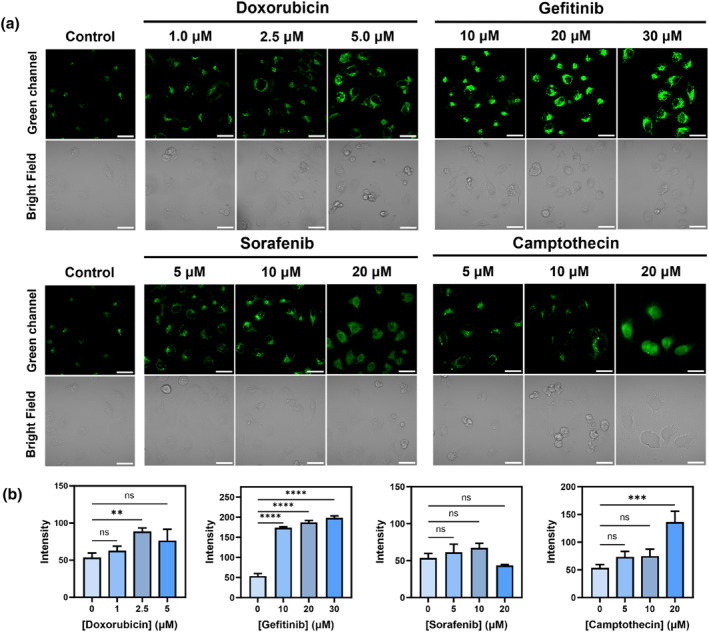
(a) Confocal laser scanning microscopy imaging of HeLa cells stained with 5 μM PMA‐H and pretreated with various concentrations of doxorubicin, gefitinib, sorafenib, and camptothecin for 12 h (*λ*_ex_ = 488 nm, emission collected at 500–550 nm in the green channel). (b) Mean fluorescence intensity in the green channel corresponding to different drug groups. Error bars represent SD, *n* = 4. **p < 0.01; ***p < 0.001; ****p < 0.0001. Scale bar: 20 μm.

*Apoptosis*: Doxorubicin (Dox), an inhibitor of topoisomerase (Topo) II, was first investigated because it induces well‐defined cell death pathways, including apoptosis.^[36]^ As the Dox concentration increased, more apoptotic cells were observed in bright‐field images, along with cell membrane shrinkage and increased lysosomal viscosity (Figure 4). In the green channel, the mean fluorescence intensity increased by 17% and 66% in the 1 and 2.5 μM Dox groups, respectively, indicating increased lysosomal viscosity. However, the mean intensity decreased in the 5 μM Dox group, in which severe apoptosis was evident in bright‐field images. Fluorescence became dimmer in regions farther from the nucleus. Overall, these observations are consistent with previous reports. In p53‐inactivated HeLa cells, Dox‐induced Topo II poisoning resulted in extensive DNA double‐strand breaks, leading to MOMP (mitochondrial outer membrane permeabilization) and LMP. These events caused efflux of small molecules and ions and increased lysosomal viscosity.[^19^, ^37^, ^38^, ^39^] In later stages of apoptosis, severe LMP disrupted lysosomal membranes, released lysosomal contents, and reduced lysosome number.[^40^, ^41^] PMA‐H in Dox‐treated HeLa cells enabled lysosomal labeling at specific stages of apoptosis and verified that lysosomal viscosity increased while lysosome number decreased.

Gefitinib (Gef) is a tyrosine kinase inhibitor of epidermal growth factor receptors.^[42]^ Recent studies have shown that Gef induces caspase‐dependent apoptosis in HeLa cells.^[43]^ As shown in Figure 4b, Gef‐treated cells exhibited markedly higher fluorescence than the control group, with increases of 224%, 248%, and 270% in the 10, 20, and 30 μM groups, respectively. In addition, dark regions in bright‐field images corresponded to intense fluorescence in the green channel. Overall, PMA‐H imaging revealed lysosome accumulation and a dramatic increase in lysosomal viscosity (more than fivefold) in Gef‐treated HeLa cells.

Sorafenib (Sor) is another multi‐kinase inhibitor that exhibits potent antitumor activity in a broad spectrum of human tumor xenograft models.^[44]^ In HeLa cells, Sor possibly induced apoptosis through a mitochondrial‐ and death receptor‐5‐mediated caspase‐8/caspase‐3 signaling pathway.^[45]^ In the 5 and 10 μM Sor‐treated groups, HeLa cells did not exhibit prominent differences in morphology or fluorescence intensity compared with the control group. In the 20 μM Sor‐treated group, apoptosis occurred. Nuclear membrane permeability became disordered, morphological alterations occurred, and LMP was induced, resulting in the appearance of PMA‐H in the cytoplasm, nucleus, and nucleolus, accompanied by a decrease in mean fluorescence intensity. As shown by PMA‐H imaging, Sor treatment may not cause significant changes in lysosomal viscosity during apoptosis.

Camptothecin (CPT), a classic inhibitor of Topo I, stabilizes the Topo I‐DNA cleavage complex and generates replication‐associated DNA breaks, thereby activating p53‐dependent stress responses and ultimately initiating the MOMP‐ and LMP‐mediated apoptotic pathway.^[46]^ As shown in Figure 4b, in the 5 and 10 μM CPT‐treated groups, there was no significant correlation between fluorescence intensity and CPT concentration. The 20 μM CPT‐treated group differed: cells were dead, as seen in bright‐field images, but fluorescence intensity increased markedly. Because no green fluorescent spots were observed in the cytoplasm, severe LMP was considered to have occurred. Moreover, the fluorescence intensity in the 20 μM group increased by 154%, suggesting that cytoplasmic viscosity increased dramatically. Nevertheless, these dead cells showed morphologies were different from those induced by other apoptotic drugs.

PMA‐H revealed changes in lysosomal viscosity, morphology, and abundance in HeLa cells after treatment with four apoptosis‐inducing drugs. Low doses of Dox, Sor, and CPT did not significantly alter lysosomal viscosity or lysosome number in HeLa cells; however, lysosomal viscosity in Gef‐treated cells increased up to 270%.

**FIGURE 5:**
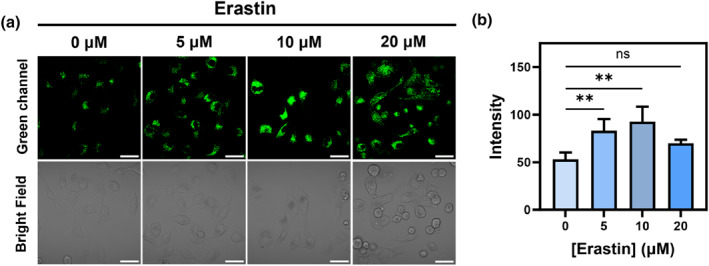
(a) Confocal laser scanning microscopy images of HeLa cells stained with 5 μM PMA‐H after pretreatment with various concentrations of erastin for 12 h (*λ*_ex_ = 488 nm; emission collected at 500–550 nm in the green channel). (b) Mean fluorescence intensity in the green channel for the erastin‐treated groups across concentrations. Error bars represent SD, *n* = 4. **p < 0.01. Scale bar: 20 μm.

*Ferroptosis*: Erastin‐induced ferroptosis in HeLa cells driven by glutathione depletion and subsequent lipid peroxidation has been widely reported and provides a robust platform for investigating lysosome‐associated biophysical changes during non‐apoptotic cell death. Recent mechanistic studies further demonstrate that lysosomal iron homeostasis and lysosomal lipid peroxidation can trigger ferroptotic cell death, highlighting a critical role for lysosomes in ferroptosis pathways.[^47^, ^48^, ^49^] Here, the role of lysosomes in erastin‐induced ferroptosis in HeLa cells was explored by imaging with PMA‐H. As shown in Figure 5a, fluorescence intensity increased in the 5 and 10 μM erastin groups, and regions with intense fluorescence in the green channel corresponded to distinct dark areas in bright‐field images. As shown in Figure 5b, the fluorescence intensity increased by 55% and 73% in the 5 and 10 μM groups, respectively. However, as erastin concentration increased, the number of ferroptotic cells in the bright‐field field of view also increased. In the 20 μM group, a large number of cells became spherical in morphology and the vesicular lysosomal structure disappeared, indicating the emergence of LMP and an attenuation of mean fluorescence intensity.^[50]^ Overall, PMA‐H demonstrated lysosome accumulation and increased lysosomal viscosity in erastin‐treated HeLa cells.

**FIGURE 6:**
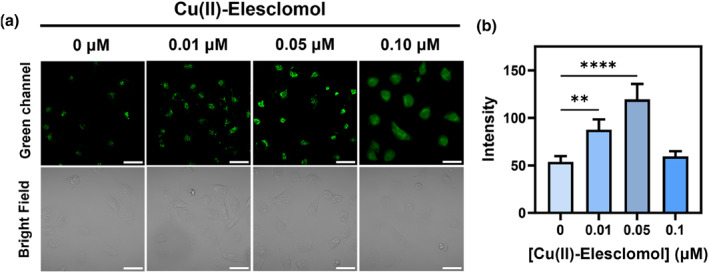
(a) Confocal laser scanning microscopy images of HeLa cells stained with 5 μM PMA‐H after pretreatment with various concentrations of Cu(II)‐elesclomol for 12 h (*λ*_ex_ = 488 nm; emission collected at 500–550 nm in the green channel). (b) Mean fluorescence intensity in the green channel for the Cu(II)‐elesclomol‐treated groups across concentrations. Error bars represent SD, *n* = 4. **p < 0.01; ****p < 0.0001. Scale bar: 20 μm.

*Cuproptosis*: Cu(II)‐elesclomol is a well‐known mitochondrion‐targeting cuproptosis drug that induces proteotoxic stress by directly binding copper to lipoylated components of the tricarboxylic acid cycle.[^51^, ^52^, ^53^, ^54^] Here, PMA‐H showed fluorescence intensity increases of 63% and 123% in the 0.01 and 0.05 μM Cu(II)‐elesclomol groups, despite similar bright‐field morphology in HeLa cells (Figure 6). This might indicate lysosomal membrane dysfunction, leakage of small molecules, and as a result, a consequent sharp increase in viscosity. However, at higher drug concentrations, Cu(II)‐elesclomol‐treated cells had the potential to suffer from disordered permeability of the nuclear membrane, morphological alterations, and LMP. In the 0.1 μM group, green‐channel images showed PMA‐H fluorescence in the cytoplasm and nucleus, including the nucleolus, with reduced mean fluorescence intensity, suggesting that lysosomal contents leaked into multiple membrane‐bound organelles. In bright‐field images, filopodia between cells disappeared, and cell membranes appeared abnormal and dysfunctional, consistent with cell death.

**FIGURE 7:**
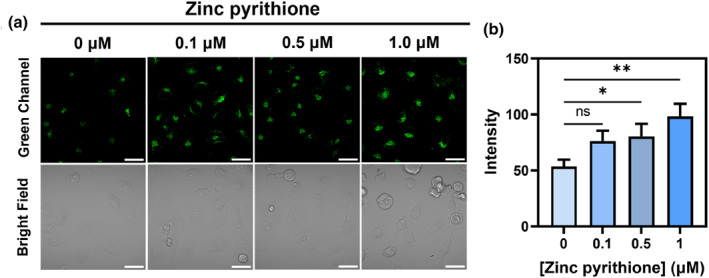
(a) Confocal laser scanning microscopy images of HeLa cells stained with 5 μM PMA‐H after pretreatment with various concentrations of zinc pyrithione for 12 h (*λ*_ex_ = 488 nm; emission collected at 500–550 nm in the green channel). (b) Mean fluorescence intensity of PMA‐H‐stained HeLa cells for the ZnPT‐treated groups across concentrations. Data were expressed as mean ± standard errors (*n* = 4). *p < 0.1; **p < 0.01. Scale bar: 20 μm.

*Zinc‐induced cell death*: Zinc pyrithione (ZnPT) is a 1:2 complex of a zinc atom and the membrane‐permeable ionophore pyrithione. It has antitumor activity in HeLa cells by promoting Zn^2+^ overload–induced lysosome‐mitochondrial apoptosis.[^55^, ^56^] Across ZnPT concentrations from 0 to 1.0 μM, the fluorescence intensity of PMA‐H‐stained HeLa cells increased monotonically with concentration (Figure 7a). Fluorescence intensity increased by 50% and 83% in the 0.5 and 1 μM groups, respectively (Figure 7b). Although bright‐field morphology changes were not obvious, green‐channel imaging showed lysosomal accumulation and increased viscosity, implying lysosomal dysfunction. Accordingly, as the concentration of ZnPT increased from 0 to 0.1 μM, cell morphology changed little, but PMA‐H was able to visualize increased lysosomal viscosity during zinc‐induced cell death.

To further verify the precise localization of PMA‐H during chemotherapy, PMA‐H was co‐stained with a commercial lysosome tracker after treatment with chemotherapeutic drugs, including Dox, Gef, erastin, Cu(II)‐elesclomol, and ZnPT. PMA‐H showed strong fluorescence overlap with the commercial lysosome tracker (Figure S13). The high PCC values (>0.9) indicated that the fluorescence enhancement of PMA‐H mainly originated from lysosomes rather than nonspecific intracellular accumulation. These results demonstrated that PMA‐H retains excellent lysosome‐targeting ability in different chemotherapy pathways, supporting its reliability for monitoring chemotherapy‐induced lysosomal viscosity changes.

## 3 CONCLUSION

In summary, a series of ultra‐sensitive viscosity‐responsive fluorescent probes (PMA‐X) was developed for subcellular monitoring of lysosomal viscosity. Owing to TICT‐regulated emission enabled by multiple rotatable aromatic units, the probes exhibit quenched fluorescence in low‐viscosity environments and markedly enhanced emission under high‐viscosity conditions. PMA‐H was identified as the optimal probe, showing a broad linear response range (0.54–1410 cP) with a 187‐fold fluorescence enhancement and high stability. PMA‐H selectively targets lysosomes and enables real‐time visualization of viscosity changes during anticancer drug–induced cell death, including apoptosis, ferroptosis, cuproptosis, and zinc‐induced cell death. Notably, in the gefitinib‐induced apoptotic model, lysosomal viscosity increased by approximately 270% compared with the control, a result markedly different from that observed with other apoptosis‐inducing agents. In both ferroptosis and cuproptosis processes, lysosomal viscosity also exhibited a moderate increase. In contrast, changes in lysosomal viscosity were not pronounced during zinc‐induced cell death, suggesting close correlations with lysosomal dysfunction and apoptotic progression. Overall, we established a new viscosity‐responsive sensing platform and provided a powerful analytical tool to quantitatively probe changes in lysosomal viscosity across diverse cell death pathways.

## CONFLICT OF INTEREST STATEMENT

The authors declare no conflicts of interest.

## ETHICS STATEMENT

No animal or human experiments were involved in this study.

## Supporting information

Supporting Information S1

## Data Availability

The data that supports the findings of this study are available in Supplementary Information S1 of this article or available from the corresponding authors on reasonable request.

## References

[1] M. Wu , M. Zhang , Y. Zhang , Z. Li , X. Li , Z. Liu , H. Liu , X. Li , Cell Death Dis. 2021, 12, 958. 34663802 10.1038/s41419-021-04271-w PMC8523726

[2] S. R. Bonam , F. Wang , S. Muller , Nat. Rev. Drug Discovery 2019, 18, 923 31477883 10.1038/s41573-019-0036-1 PMC7097195

[3] Y. Xing , M.‐M. Wang , F. Zhang , T. Xin , X. Wang , R. Chen , Z. Sui , Y. Dong , D. Xu , X. Qian , Q. Lu , Q. Li , W. Cai , M. Hu , Y. Wang , J.‐L. Cao , D. Cui , J. Qi , W. Wang , Nat. Commun. 2025, 16, 985. 39856099 10.1038/s41467-025-56403-x PMC11760952

[4] J. Feng , Z.‐X. Wang , J.‐L. Bin , Y.‐X. Chen , J. Ma , J.‐H. Deng , X.‐W. Huang , J. Zhou , G.‐D. Lu , Cancer Lett. 2024, 587, 216728. 38431036 10.1016/j.canlet.2024.216728

[5] X. Li , R. Zhao , Y. Wang , C. Huang , J. Mater. Chem. B 2018, 6, 6592. 32254867 10.1039/c8tb01885e

[6] X. Chao , Y. Hu , R. Liu , D. Huang , Y. Zhang , Sens. Actuators, B 2021, 345, 130397.

[7] Y. Lee , H. Jeong , H. Bian , Y. Li , J. Park , H. Xu , J. Yoon , Sens. Actuators, B 2026, 449, 139167.

[8] K. Kwapiszewska , K. Szczepański , T. Kalwarczyk , B. Michalska , P. Patalas‐Krawczyk , J. Szymański , T. Andryszewski , M. Iwan , J. Duszyński , R. Hołyst , J. Phys. Chem. Lett. 2020, 11, 6914. 32787203 10.1021/acs.jpclett.0c01748 PMC7450658

[9] A. T. Molines , J. Lemière , M. Gazzola , I. E. Steinmark , C. H. Edrington , C.‐T. Hsu , P. Real‐Calderon , K. Suhling , G. Goshima , L. J. Holt , M. Thery , G. J. Brouhard , F. Chang , Dev. Cell 2022, 57, 466. 35231427 10.1016/j.devcel.2022.02.001 PMC9319896

[10] S. Qin , X. Cheng , Z. Zhou , X. Zhang , J. Chen , P. Xu , T. Wu , Y. Hu , ACS Nano 2025, 19, 24985. 40590707 10.1021/acsnano.5c04279

[11] T. Su , R. Shen , D. Tu , X. Han , X. Luo , F. Yu , Smart Mol. 2025, 3, e20240062. 40625559 10.1002/smo.20240062 PMC12117884

[12] F. Bonacina , X. Zhang , N. Manel , L. Yvan‐Charvet , B. Razani , G. D. Norata , Nat. Rev. Cardiol. 2025, 22, 149. 39304748 10.1038/s41569-024-01072-4 PMC11835540

[13] F. Gros , S. Muller , Nat. Rev. Nephrol. 2023, 19, 366. 36894628 10.1038/s41581-023-00692-2

[14] P. Wang , S. Ai , M. Deng , Y. Liu , Y. Liu , L. He , S. Li , Talanta 2024, 278, 126506. 38968659 10.1016/j.talanta.2024.126506

[15] C. Vrancx , W. Annaert , Nat. Cell Biol. 2025, 27, 553. 40140604 10.1038/s41556-024-01608-3

[16] L. Pan , H. Peng , B. Lee , J. Zhao , X. Shen , X. Yan , Y. Hua , J. Kim , D. Kim , M. Lin , S. Zhang , X. Li , X. Yi , F. Yao , Z. Qin , J. Du , Y. Chi , J.‐M. Nam , T. Hyeon , J. Liu , Adv. Mater. 2024, 36, 2305394. 10.1002/adma.202305394 37643367

[17] N. Fehrenbacher , M. Jäättelä , Cancer Res. 2005, 65, 2993. 15833821 10.1158/0008-5472.CAN-05-0476

[18] X. Wu , L. Cao , Q. Zhao , J. Kou , Y. Li , F. Kong , B. Tang , Chem. Commun. 2025, 61, 17902. 10.1039/d5cc04607f 41114717

[19] G. Yamashita , N. Takano , H. Kazama , K. Tsukahara , K. Miyazawa , Cell Death Discovery 2022, 8, 502. 36581628 10.1038/s41420-022-01293-x PMC9800408

[20] M. Grayson , Nature 2016, 537, S145. 27652778 10.1038/537S145a

[21] H. Bian , D. Ma , X. Zhang , Y. Qiu , X. Wu , M. Jia , X. Zhang , X. Liu , Y. Yang , X. Peng , J. Yoon , Y. Xiao , J. Am. Chem. Soc. 2025, 147, 39936. 41088764 10.1021/jacs.5c15490

[22] Y.‐Y. Zhao , B. Hwang , Y. Lee , J. Yoon , Natl. Sci. Rev. 2024, 11, nwae048. 38405433 10.1093/nsr/nwae048 PMC10894028

[23] Y.‐Y. Zhao , Y. Xu , J. Wen , H. Jeong , H. Kim , X. Li , J. Yoon , J. Am. Chem. Soc. 2025, 147, 48072. 41241839 10.1021/jacs.5c14960

[24] Y.‐Y. Zhao , X. Zhang , Y. Xu , Z. Chen , B. Hwang , H. Kim , H. Liu , X. Li , J. Yoon , Angew. Chem., Int. Ed. 2024, 63, e202411514. 10.1002/anie.202411514 38940633

[25] M. Yang , Y. Kim , S.‐Y. Youn , H. Jeong , M. E. Shirbhate , C. Uhm , G. Kim , K. T. Nam , S.‐S. Cha , K. M. Kim , J. Yoon , Biomaterials 2025, 313, 122792. 39226652 10.1016/j.biomaterials.2024.122792

[26] H. Xu , L. Lu , C. Huang , L. Liu , H. Bian , H. Zhang , J. Yoon , CCS Chem. 2026, 8, 1924.

[27] Y. Li , M. Wang , F. Wang , S. Lu , X. Chen , Smart Mol. 2023, 1, e20220003. 40625648 10.1002/smo.20220003 PMC12118285

[28] D. Li , C. Lu , S. Sun , X. Li , Y. Xiao , X. Zhang , Smart Mol. 2025, 3, e70015. 41035513 10.1002/smo2.70015 PMC12483125

[29] X.‐Z. Yang , S. Yao , J. Wu , J. Diao , W. He , Z. Guo , Y. Chen , Smart Mol. 2024, 2, e20240040. 40626270 10.1002/smo.20240040 PMC12118280

[30] L. Wu , Y. Wang , T. D. James , N. Jia , C. Huang , Chem. Commun. 2018, 54, 5518. 10.1039/c8cc02330a 29670954

[31] M. Dong , D. Wang , J. Yang , P. Sun , W. Ding , J. Yang , J. Yan , W. Chi , Smart Mol. 2023, 1, e20230011. 40626206 10.1002/smo.20230011 PMC12118249

[32] B. Sun , L. Liu , J. Yoon , J. Phys. Chem. A 2025, 129, 2420. 40011218 10.1021/acs.jpca.4c07483

[33] L. Hou , P. Ning , Y. Feng , Y. Ding , L. Bai , L. Li , H. Yu , X. Meng , Anal. Chem. 2018, 90, 7122. 29865790 10.1021/acs.analchem.8b01631

[34] C. Liu , T. Zhao , S. He , L. Zhao , X. Zeng , J. Mater. Chem. B 2020, 8, 8838. 33026403 10.1039/d0tb01329c

[35] L. Wang , Y. Xiao , W. Tian , L. Deng , J. Am. Chem. Soc. 2013, 135, 2903. 23409947 10.1021/ja311688g

[36] E. Christidi , L. R. Brunham , Cell Death Dis. 2021, 12, 339. 33795647 10.1038/s41419-021-03614-x PMC8017015

[37] Y. Jiang , Y. Liu , W. Xiao , D. Zhang , X. Liu , H. Xiao , S. You , L. Yuan , Oxid. Med. Cell. Longevity 2021, 2021, 5896931. 10.1155/2021/5896931 PMC8019640 33854694

[38] C.‐P. Lin , S.‐H. Wu , T.‐Y. Lin , C.‐H. Chu , L.‐W. Lo , C.‐C. Kuo , J.‐Y. Chang , S.‐C. Hsu , B.‐S. Ko , M. Yao , J.‐K. Hsiao , S.‐W. Wang , D.‐M. Huang , Pharmacol. Res. 2023, 197, 106945. 37797662 10.1016/j.phrs.2023.106945

[39] S. Kiraly , J. Stanley , E. R. Eden , Antioxidants 2025, 14, 125. 40002312 10.3390/antiox14020125 PMC11852311

[40] P. Boya , K. Andreau , D. Poncet , N. Zamzami , J.‐L. Perfettini , D. Metivier , D. M. Ojcius , M. Jäättelä , G. Kroemer , J. Exp. Med. 2003, 197, 1323. 12756268 10.1084/jem.20021952 PMC2193790

[41] K. S. Allemailem , A. Almatroudi , F. Alrumaihi , S. A. Almatroodi , M. O. Alkurbi , G. T. Basfar , A. H. Rahmani , A. A. Khan , Int. J. Nanomed. 2021, 16, 5065. 10.2147/IJN.S321343 PMC8324981 34345172

[42] M. Tiseo , M. Bartolotti , F. Gelsomino , P. B. P , Drug Des., Dev. Ther. 2010, 4, 81. 10.2147/dddt.s6594 PMC2880339 20531963

[43] B. C. Jung , S.‐H. Woo , S. H. Kim , Y. S. Kim , BMB Rep. 2024, 57, 104. 38303562 10.5483/BMBRep.2023-0225 PMC10910092

[44] L. Adnane , P. A. Trail , I. Taylor , S. M. Wilhelm , Methods Enzymol. 2006, 407, 597. 16757355 10.1016/S0076-6879(05)07047-3

[45] M.‐T. Lin , C.‐L. Lin , T.‐Y. Lin , C.‐W. Cheng , S.‐F. Yang , C.‐L. Lin , C.‐C. Wu , Y.‐H. Hsieh , J.‐P. Tsai , Tumor Biol. 2016, 37, 6987. 10.1007/s13277-015-4526-4 26662956

[46] Y. Pommier , Chem. Rev. 2009, 109, 2894. 19476377 10.1021/cr900097c PMC2707511

[47] J. Wang , N. Wu , M. Peng , L. Oyang , X. Jiang , Q. Peng , Y. Zhou , Z. He , Q. Liao , Cell Death Discovery 2023, 9, 463. 38110359 10.1038/s41420-023-01753-y PMC10728094

[48] T. Cañeque , L. Baron , S. Müller , A. Carmona , L. Colombeau , A. Versini , S. Solier , C. Gaillet , F. Sindikubwabo , J. L. Sampaio , M. Sabatier , E. Mishima , A. Picard‐Bernes , L. Syx , N. Servant , B. Lombard , D. Loew , J. Zheng , B. Proneth , L. K. Thoidingjam , L. Grimaud , C. S. Fraser , K. J. Szylo , E. Der Kazarian , C. Bonnet , E. Charafe‐Jauffret , C. Ginestier , P. Santofimia‐Castaño , M. Estaras , N. Dusetti , J. L. Iovanna , A. S. Cunha , G. Pittau , P. Hammel , D. Tzanis , S. Bonvalot , S. Watson , V. Gandon , A. Upadhyay , D. A. Pratt , F. P. Freitas , J. P. Friedmann Angeli , B. R. Stockwell , M. Conrad , J. M. Ubellacker , R. Rodriguez , Nature 2025, 642, 492.40335696

[49] Z. Shi , K. Chen , Y. Wang , H. Du , Cell. Mol. Neurobiol. 2025, 45, 73. 40684405 10.1007/s10571-025-01593-7 PMC12277238

[50] Y. Saimoto , D. Kusakabe , K. Morimoto , Y. Matsuoka , E. Kozakura , N. Kato , K. Tsunematsu , T. Umeno , T. Kiyotani , S. Matsumoto , M. Tsuji , T. Hirayama , H. Nagasawa , K. Uchida , S. Karasawa , M. Jutanom , K.‐i. Yamada , Nat. Commun. 2025, 16, 3554. 40229298 10.1038/s41467-025-58909-w PMC11997074

[51] M. Nagai , N. H. Vo , L. Shin Ogawa , D. Chimmanamada , T. Inoue , J. Chu , B. C. Beaudette‐Zlatanova , R. Lu , R. K. Blackman , J. Barsoum , K. Koya , Y. Wada , Free Radical Biol. Med. 2012, 52, 2142. 22542443 10.1016/j.freeradbiomed.2012.03.017

[52] P. Tsvetkov , S. Coy , B. Petrova , M. Dreishpoon , A. Verma , M. Abdusamad , J. Rossen , L. Joesch‐Cohen , R. Humeidi , R. D. Spangler , J. K. Eaton , E. Frenkel , M. Kocak , S. M. Corsello , S. Lutsenko , N. Kanarek , S. Santagata , T. R. Golub , Science 2022, 375, 1254. 35298263 10.1126/science.abf0529 PMC9273333

[53] H. Bian , D. Ma , Y. Chen , J. Hong , Y. Nan , H. Xu , M. H. Kim , X. Chen , X. Peng , J. Yoon , Chem. Soc. Rev. 2026, 55, 3139. 41685519 10.1039/d5cs01306b

[54] D. Ma , H. Bian , F. Pan , D. Zhou , Z. Chen , H. Ge , Y. Guo , Y. Wu , X. He , P. Zhou , L. Wang , X. Chen , X. Peng , Chem. Sci. 2026, 17, 8061. 41767800 10.1039/d5sc08871b PMC12947697

[55] M. Chen , Y. Ding , Y. Ke , Y. Zeng , N. Liu , Y. Zhong , X. Hua , Z. Li , Y. Xiong , C. Wu , H. Yu , Artif. Cells, Nanomed., Biotechnol. 2020, 48, 824. 32456481 10.1080/21691401.2020.1770266

[56] H. Fang , S. Geng , M. Hao , Q. Chen , M. Liu , C. Liu , Z. Tian , C. Wang , T. Takebe , J.‐L. Guan , Y. Chen , Z. Guo , W. He , J. Diao , Nat. Commun. 2021, 12, 109. 33397937 10.1038/s41467-020-20309-7 PMC7782730

