# Supplementary material for: A lysosome‐targeted ultra‐sensitive viscosity probe for monitoring viscosity alterations during chemotherapy
Source: Smart Mol. 2026 Jun 10:e70070. Online ahead of print. doi: 10.1002/smo2.70070 (PMC13398979; doi:10.1002/smo2.70070)
Supplement: Supplementary file 1 — Supporting Information S1 [file SMO2-9999-0-s001.docx]

Supporting Information

A Lysosome-Targeted Ultra-Sensitive Viscosity Probe for Monitoring Viscosity Alterations During Chemotherapy

*Haoyang Song^a, c^, Hui Bian^a, d,^*, Lei Liu^b^, Gahyun Park^a, c^, Bingqing Sun^b^, Qiongzheng Hu^e, f,^*, and Juyoung Yoon^a, c,^**

^a^ Department of Chemistry and Nanoscience, Ewha Womans University, Seoul 03760, Republic of Korea.

^b^ College of Chemistry and Materials Engineering, Anhui Science and Technology University, Bengbu 233000, China.

^c^ Graduate Program in Innovative Biomaterials Convergence, Ewha Womans University, Seoul 03760, Republic of Korea.

^d^ New and Renewable Energy Research Center, Ewha Womans University, Seoul 03760, Republic of Korea.

^e^ School of Pharmaceutical Sciences, Qilu University of Technology (Shandong Academy of Sciences), Jinan 250014, China.

^f^ Qilu University of Technology (Shandong Academy of Sciences), Shandong Analysis and Test Center, Jinan 250014, China.

Table of Content

1. **Instruments and Experimental Section**
2. **Synthesis methods of each compound and final products**
3. **Synthesis of the Cu(II)-elesclomol**
4. **Optical performance of PMA-X**
5. **Measurement of fluorescence quantum yields (ф)**
6. **Cell culture**
7. **Cytotoxicity assays**
8. **CLSM imaging**

**Table S1.** Recent viscosity-response fluorescence probes and comparations of their properties.

**Table S2.** The oscillator strength of **PMA-H** at different dihedral angles.

**Figure S1.** The synthesis routes of **PMA-X** and their mechanism of viscosity response luminescence.

**Figure S2.** Absorbance spectra and fluorescence spectra of **PMA-Br** and **PMA-I** (5 μM) in various organic solvents.

**Figure S3.** Optimized conformation of **PMA-H** in S_0_ state and their orbitals.

**Figure S4.** Optimized conformation of **PMA-H** in S_1_ state and their orbitals.

**Figure S5.** Torsional potential energy of **PMA-H** with different dihedral angles.

**Figure S6.** Maximum absorption and maximum fluorescence emission spectrum of **PMA-X**.

**Figure S7.** Absorbance spectra and fluorescence spectra of **PMA-Br** and **PMA-I** (5 μM) in various aqueous conditions.

**Figure S8.** Fluorescence intensity of **PMA-X** in THF and mixture of THF and water.

**Figure S9.** Fluorescence intensity of **PMA-Br** an **PMA-I** in methanol/glycerol mixtures with viscosity increase.

**Figure S10.** The relative absorbance and fluorescence intensity of **PMA-H** after different time of irradiation.

**Figure S11.** Cytotoxicity of **PMA-X**.

**Figure S12.** CLSM imaging of HeLa with different incubation time of **PMA-H**, and their mean fluorescence intensity.

**Figure S13.** Colocalization of **PMA-H** and commercial lysosome tracker in drug-treated HeLa cells.

**Figure S14.** ^1^H NMR spectrum of compound **2** in CDCl_3_.

**Figure S15.** ^13^C NMR spectrum of compound **2** in CDCl_3_.

**Figure S16.** ^1^H NMR spectrum of **PMA-H** in CDCl_3_.

**Figure S17.** ^13^C NMR spectrum of **PMA-H** in CDCl_3_.

**Figure S18.** ^1^H NMR spectrum of **PMA-Br** in CDCl_3_.

**Figure S19.** ^13^C NMR spectrum of **PMA-Br** in CDCl_3_.

**Figure S20.** ^1^H NMR spectrum of **PMA-I** in CDCl_3_.

**Figure S21.** ^13^C NMR spectrum of **PMA-I** in CDCl_3_.

**Figure S22.** ESI-HRMS spectrum of **PMA-H**.

**Figure S23.** ESI-HRMS spectrum of **PMA-Br**.

**Figure S24.** ESI-HRMS spectrum of **PMA-I**.

**1.** **Instruments and Experimental Section**

Dimethyl sulfoxide (DMSO), toluene, ethyl acetate (EA), dichloromethane (DCM) Lawesson reagent (Michler’s ketone), 4,4′-bis(dimethylamino)benzophenone glycerol, iodomethane, aniline, 4-bromoaniline, 4-iodoaniline, diethylamine NONOate sodium salt hydrate, DNA from calf thymus, hydrogen peroxide solution (30%), cystamine dihydrochloride, D-(+)-galactose, L-glutamine, uric acid, calcium chloride, copper chloride, aluminum chloride, magnesium sulfate, ammonium iron(II) sulfate hexahydrate, sodium hypochlorite, doxorubicin, gefinitib, sorafenib, camptothecin, zinc pyrithione, erastin and elesclomol were purchased from Sigma-Aldrich Inc., Seoul, Korea. LysoTracker™ Blue DND-22, ER-Tracker™ Red (BODIPY™ TR Glibenclamide), and MitoTracker™ Deep Red FM Dye, was purchased by Thermo Fisher Scientific Inc. All other solvents and reagents were analytical grade and employed as received. Fluorescence emission and electronic absorption wavelength of these compounds were measured on a Scinco FS-2 spectrofluorometer and Jasco V-770 spectrophotometer, respectively. The 1H NMR spectrum measurement was performed using a Bruker AM 300 spectrometer (300 MHz) at the National Research Facilities and Equipment Center (NanoBio·Energy Materials Center) at Ewha Womans University. Fluorescence imaging of cells was taken by laser scanning confocal microscopy from Zeiss LSM 900. Data was treated by OriginPro 2018C 64-bit and GraphPad Prism10, besides, fluorescence imagines were deal with ImageJ2.

**2. Synthesis methods of each compound and final products**

**Compound 1:**

Lawesson reagent (186 μmol, 75.36 mg) was added to a stirred solution of Michler’s ketone (373 μmol, 100 mg) in one-pot toluene. The reactive solution was refluxed for 12 h in a nitrogen atmosphere. After the solution was cooled down into room temperature, saturated sodium bicarbonate aqueous solution was slowly and carefully added and stirred for 15 min. Before removing solvent through vacuum distillation, the organic phase was previously extracted three times with EA. The raw product was collected and dried in fume hood.

**Compound 2**:

To stirred solvent of 2 mL iodomethane, **compound 1** (176 μmol, 50 mg) which is prepared before is added. The mixture was stirred in a sealed environment filled with nitrogen for 2 h. After vacuum distillation, the mixture was purified by silica gel chromatography using Petroleum ether/ ethyl acetate (v/v, 8 : 1) as the eluent to afford product as a dark green solid.

**PMA-X**:

In light of the different substitutes of final product, aniline (499 μmol, 45.9 mg), 4-bromoaniline (499 μmol, 85.8 mg), and 4-iodoaniline (499 μmol, 109.3 mg) were added respectively to three pots of **compound 2** (99.8 μmol, 30 mg) dissolved by mixture of DCM and acetonitrile (v/v, 1 : 10). The reaction flask was heated in oil bath for 50°C overnight. After the cooling down reaction solution and evaporating solvents, the remained substance was purified by silica gel chromatography. [v(n-Hexane)/v(ethyl acetate)=5:1].

**PMA-H**: ^1^H NMR (300 MHz, Chloroform-d) δ 7.70 (d, J = 9.0 Hz, 2H), 7.23 – 7.14 (m, 2H), 7.07 (d, J = 8.9 Hz, 2H), 6.95 (t, J = 7.4 Hz, 1H), 6.89 (d, J = 7.1 Hz, 2H), 6.71 (d, J = 9.0 Hz, 2H), 6.55 (d, J = 8.9 Hz, 2H), 3.02 (d, J = 26.2 Hz, 12H). ^13^C NMR (75 MHz, Chloroform-*d*) δ 168.67, 152.39, 150.47, 132.11, 131.76, 128.54, 123.13, 122.60, 122.00, 111.18, 110.79, 40.31, 40.16. ESI-Mass: m/z calcd for C_23_H_25_N_3_, 343.2048; found 344.2127.

**PMA-Br**: ^1^H NMR (300 MHz, Chloroform-*d*) δ 7.69 – 7.61 (m, 2H), 7.48 – 7.40 (m, 2H), 7.04 – 6.96 (m, 2H), 6.69 (d, *J* = 8.9 Hz, 2H), 6.55 (dd, *J* = 8.8, 2.5 Hz, 4H), 3.02 (d, *J* = 18.4 Hz, 12H). ^13^C NMR (75 MHz, Chloroform-d) δ 169.04, 152.24, 150.34, 131.80, 131.45, 127.70, 123.62, 123.16, 115.17, 111.10, 110.84, 40.26, 40.14. ESI-Mass: m/z calcd for C_23_H_24_N_3_Br, 422.1233; found 422.1232.

**PMA-I**: ^1^H NMR (300 MHz, Chloroform-*d*) δ 7.66 (d, *J* = 9.0 Hz, 2H), 7.28 – 7.23 (m, 2H), 7.05 – 6.98 (m, 2H), 6.73 – 6.63 (m, 4H), 6.58 – 6.52 (m, 2H), 3.02 (d, *J* = 20.2 Hz, 12H). ^13^C NMR (75 MHz, Chloroform-*d*) δ 168.96, 152.24, 150.34, 137.40, 131.81, 131.47, 124.11, 123.12, 111.10, 110.84, 40.27, 40.15. ESI-Mass: m/z calcd for C_23_H_24_N_3_I, 470.1094; found 470.1093.

**3. Synthesis of the Cu(II)-elesclomol**

To a stirred solution of elesclomol (5 mg, 12.5 μmol) in ethanol (5.0 ml), CuCl_2_·was added in one portion. The mixture was stirred at room temperature for 20 min. Water was then added, and the precipitated solid was collected by filtration. The solid was dissolved in methylene chloride and washed with water (2×), dried (Na_2_SO_4_), filtered and concentrated to give the dark red solid.^[1]^

**4. Optical performance of PMA-X**

5 μM **PMA-X** were dissolved in organic and inorganic solvents for absorbance spectrum and fluorescence emission spectrum, including toluene, dichloromethane, ethyl acetate, trichloromethane, acetonitrile, methanol, dimethyl sulfoxide, glycerol, as well as water with different pH values. Secondly, solutions of glycerol and methanol with gradually increasing viscosity were prepared to test the fluorescence response of molecules to environmental viscosity. Additionally, sensitivity of **PMA-X** towards interfering substances commonly present in biological environments was tested.

**5.** **Measurement of fluorescence quantum yields (****ф)**

The steady-state absorption and excited-state fluorescence spectroscopies of **PMA-X** in glycerol were recorded at room temperature. The relative fluorescence quantum yields were determined with rhodamine 6G (ф=0.950, in ethanol) as a standard and calculated using the following equation:^[2]^

$$ф_{sam}=ф_{std}\times\frac{A_{std}}{A_{sam}}\times\frac{I_{sam}}{I_{std}}\times\frac{\eta_{sam}^{2}}{\eta_{std}^{2}}$$

Where *sam* and *std* means unknown sample need to be tested and known standard compound, which is Rhodamine 6G here. A represents the absorbance of compounds at excitation wavelength. *I* stands for integrated area of fluorescence spectrum of the same range while excited at wavelength that *A* represented. And *η* is refractive index of solvent.^[3]^ Based on the formula above, ф of **PMA-H** is 0.169, of **PMA-Br** is 0.095, of **PMA-I** is 0.008.

**6. Cell culture**

HeLa cells were cultured in Dulbecco’s Modified Eagle Medium (DMEM) supplemented with high glucose (90%), fetal bovine serum (FBS, 9%), and penicillin/streptomycin (P/S, 100 U/mL each, 1%) under standard incubation conditions of 5% CO_2_ at 37°C.

**7. Cytotoxicity assays**

3-(4,5-dimethylthiazol-2-yl)-2,5-diphenyltetrazolium bromide (MTT) assays were carried out to evaluate the toxicity of **PMA-X**. HeLa cells were seeded into 96-well microtiter plates with cell number of 10^4^ per well. After 12 h of incubation, various concentrations of PMA-X (0, 0.1, 0.5, 1, 2, 4, 6, 8, 10, 15, 20, 40 μM) were added, and then, HeLa cells were cultured for another 24 h in dark. Afterwards, 20 μL of MTT solution (5 mg/mL) was added to each well. After 4 h, the MTT solution was removed, and 100 μL of DMSO was added to each well. Finally, the absorbance at 570 nm was measured using microplate reader.

**8. CLSM imaging**

In uptake time and stability experiments, HeLa cells were incubated with the probe **PMA-H** (5 µM) for different times (from 0.5 h to 12 h) to investigate the biocompatibility and stability of **PMA-H** in physiological microenvironment. For the lysosome colocalization imaging, HeLa cells were incubated with **PMA-H** for 30 min and with Lysotracker, ERTracker, and MitoTracker for 15 min, respectively. For the lysosome dynamic tracking imaging, HeLa cells were pretreated with anti-tumor compounds in different concentrations for 12 h and then incubated with **PMA-H** (5 µM) for 30 min before imaging.

**Table S1.** Recent viscosity-response fluorescence probes and comparations of their properties.

| Probe | Target | Emission peak | Detection range (cP) | fluorescence enhancement | Fluorescence quantum yield (*ф*) | Ref. |
| --- | --- | --- | --- | --- | --- | --- |
| L1C | Lysosome | 585 nm | 3.0 to 326.0 | 8-fold | 0.185 (95% glycerol) | [4] |
| Lyso-V | Lysosome | 517 nm | 0.6 to 359.6 | 10-fold | 0.21 (48 cP) | [5] |
| NP-V | Lysosome | 864 nm | 9.2 to 460.0 | 13-fold | 0.34 (100% glycerol) | [6] |
| CBI-V | Mitochondron | 610 nm | 1.4 to 956 | 32-fold | 0.293 (100% glycerol) | [7] |
| Lyso-Vis | Lysosome | 672 nm | 1.8 to 178 | 42.5-fold | N/A | [8] |
| Mito-BCy | Mitochondron | 635 nm | 0.89 to 945 | 68.8-fold | 0.168 (100% glycerol) | [9] |
| MQA-DNP | Mitochondron | 740 nm | 0.89 to 163.6 | 115-fold | 0.04904 (99% glycerol) | [10] |
| Lyso-QAP3 | Lysosome | 640 nm | 1.5 to 258.1 | N/A | 0.2567 (100% glycerol) | [11] |
| PMA-H | Lysosome | 565 nm | 0.54 to 1410 | 187-fold | 0.169 (100% glycerol) | This work |

**Table S2.** The oscillator strength of **PMA-H** at different dihedral angles.

| Degree of dihedral angle (°) | Oscillator strength |
| --- | --- |
| 90 | 0.0007 |
| 80 | 0.0092 |
| 70 | 0.0396 |
| 60 | 0.1093 |
| 50 | 0.2019 |
| 40 | 0.2847 |
| 30 | 0.3356 |
| 20 | 0.3423 |
| 10 | 0.2850 |
| 0 | 0.1530 |


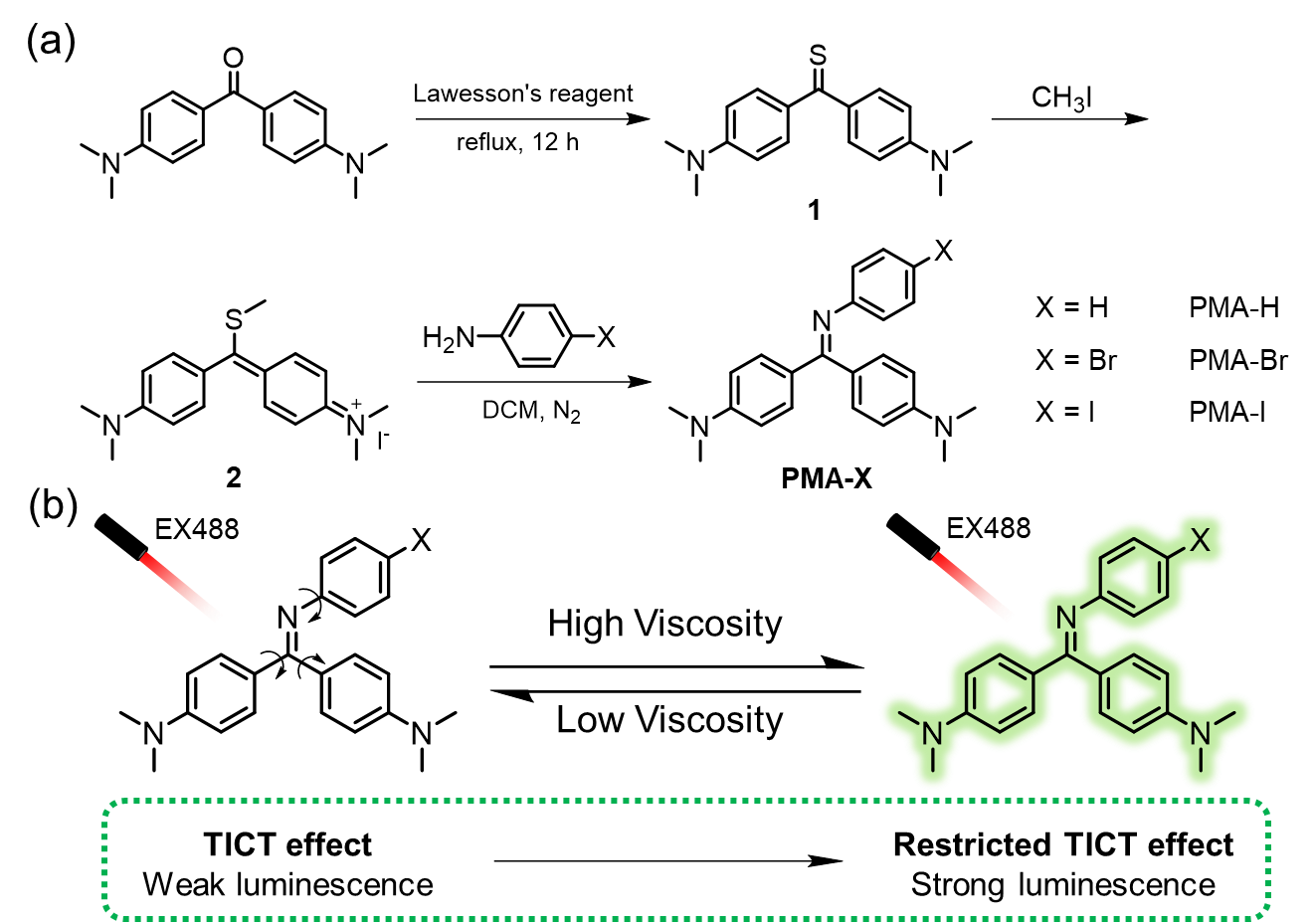


**Figure S1.** (a) The synthesis routes of **PMA-X** and (b) their mechanism of viscosity response luminescence.


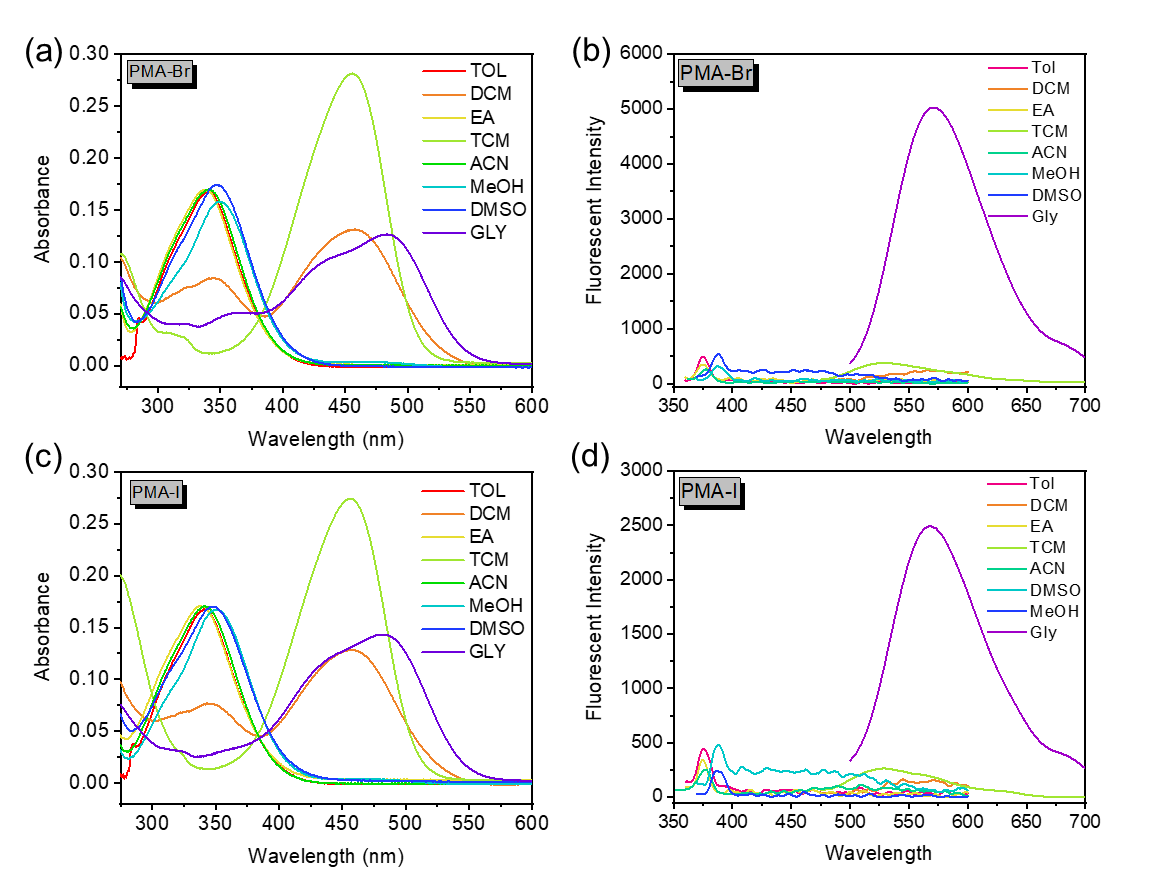


**Fugure S2.** (a)Absorbance spectra and (b) fluorescence spectra of **PMA-Br** (5 μM) in various organic solvents. And (c)absorbance spectra and (d) fluorescence spectra of **PMA-I** (5 μM) in various organic solvents. The solvents include toluene (Tol), dichloromethane (DCM), ethyl acetate (EA), trichloromethane (TCM), acetonitrile (ACN), methanol (MeOH), dimethyl sulfoxide (DMSO) and glycerol (Gly).


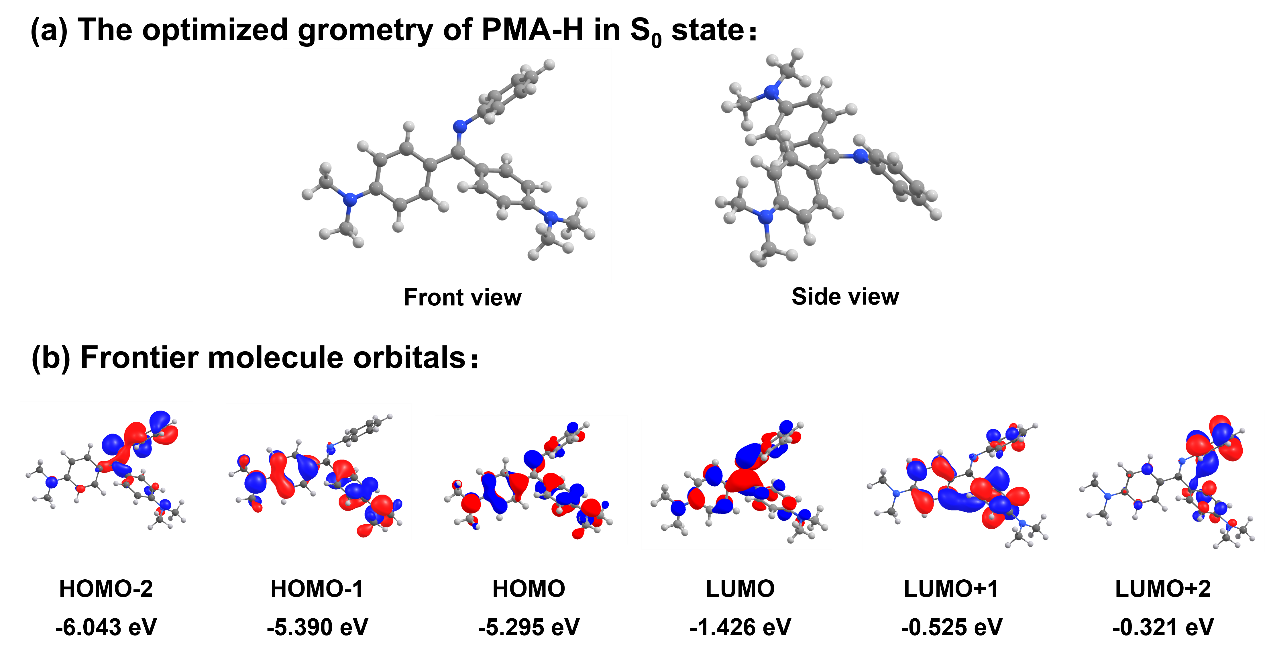


**Figure S3.** (a) Optimized conformation of **PMA-H** in S_0_ state. (b) Frontier molecular orbitals of S_0_-state PMA-H in HOMO-2, HOMO-1, HOMO, LUMO, LUMO+1, LUMO+2 orbitals.


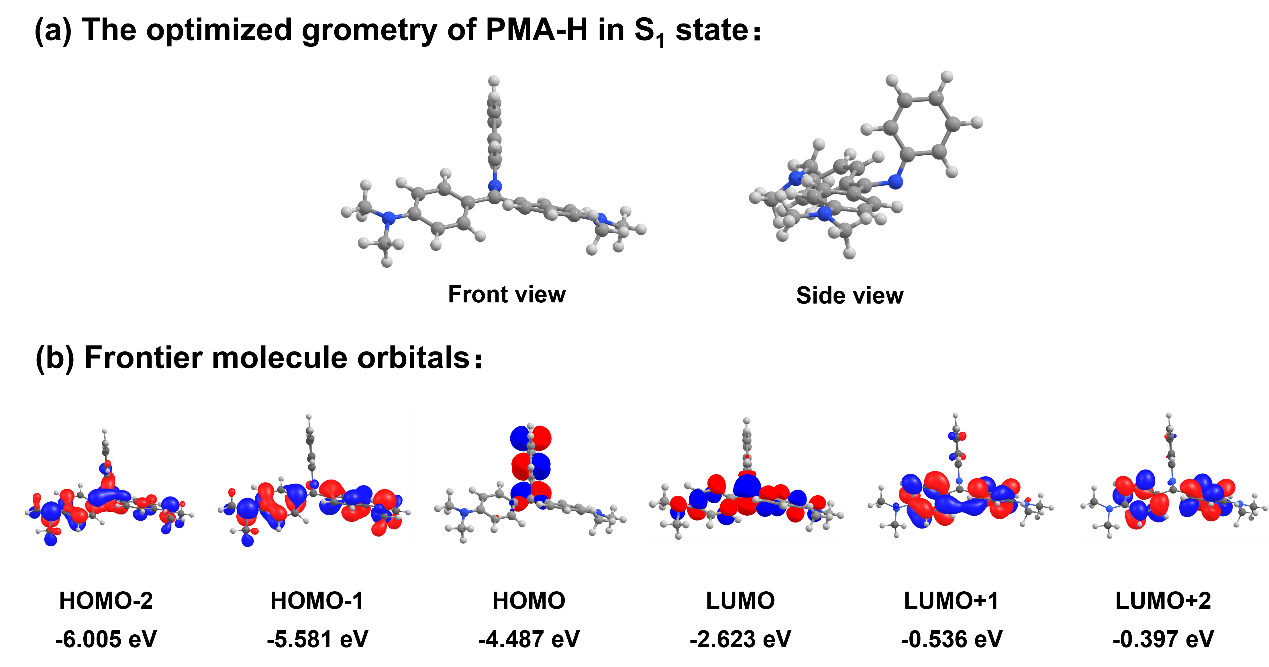


**Figure S4.** (a) Optimized conformation of **PMA-H** in S_1_ state. (b) Frontier molecular orbitals of S_1_-state **PMA-H** in HOMO-2, HOMO-1, HOMO, LUMO, LUMO+1, LUMO+2 orbitals.

**Figure S5.** Torsional potential energy of **PMA-H** with different dihedral angles.


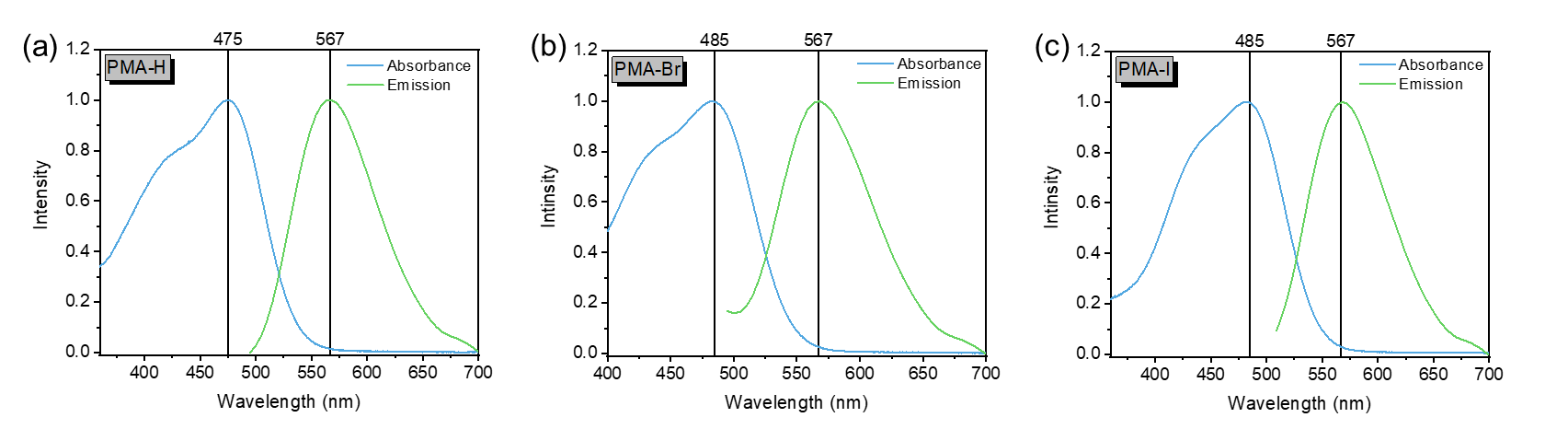


**Figure S6.** Absorption and fluorescence emission spectrum of (a) **PMA-H**, (b) **PMA-Br**, and (c) **PMA-I**, with the wavelength of maximum absorption and emission marked on upper axis.

**Figure S7.** Absorbance spectrum of (a) **PMA-Br** and (b) **PMA-I** (5 μM) in PBS buffer containing 5 mg/mL HSA, and PBS buffer with pH value 10.0, 7.4, and 4.0. And fluorescence spectrum of (c) **PMA-Br** and (d) **PMA-I** (5 μM) in PBS buffer containing 5 mg/mL HSA, and PBS buffer with pH value 10.0, 7.4, and 4.0.

**Figure S8.** The fluorescence intensity of **PMA-X** in 100% tetrahydrofuran (THF), mixture of 90% pure water and 10% THF, and 100% Gly. Data were expressed as mean±standard errors (*n*=3).


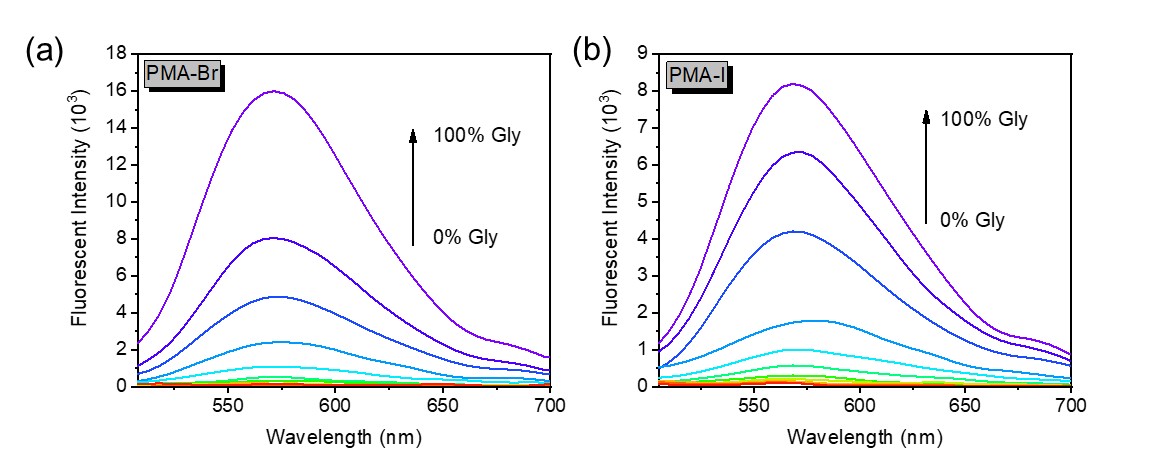


**Figure S9.** Fluorescence intensity of (a) **PMA-Br** and (b) **PMA-I** (5 μM) in methanol/glycerol mixtures with viscosity increase.

**Figure S10.** (a) The relative absorbance at 475 nm and (b) fluorescence intensity of **PMA-H** at 567 nm, after different time of irradiation (460 nm, 5 mW/cm^2^).

**Figure S11.** Cytotoxicity of **PMA-H**, **PMA-Br**, and **PMA-I** towards HeLa cell with an incubation time of 24 h. Data were expressed as mean±standard errors (*n*=6).


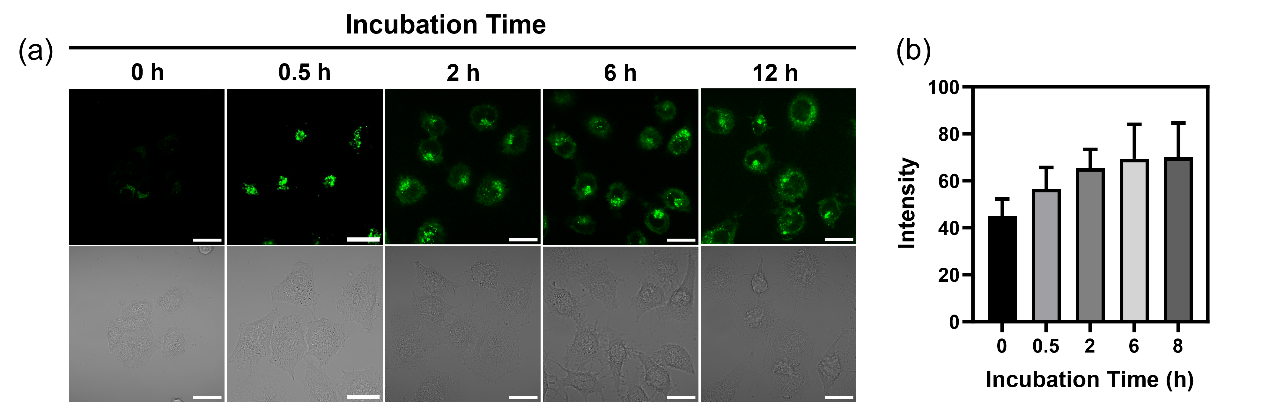


**Figure S12.** Experiment of HeLa cells taking up **PMA-H**. (a) Fluorescence and bright field imaging of HeLa cells, which were treated with **PMA-H** (5 μM) and then cultured in incubator for different time. (Green channel: λex=488 nm, λem from 500 to 550 nm) (b) Mean fluorescence intensity of **PMA-H** treated HeLa cells corresponding to different incubation time. Data were expressed as mean±standard errors (*n*=3). Scale bar is 20 μm.


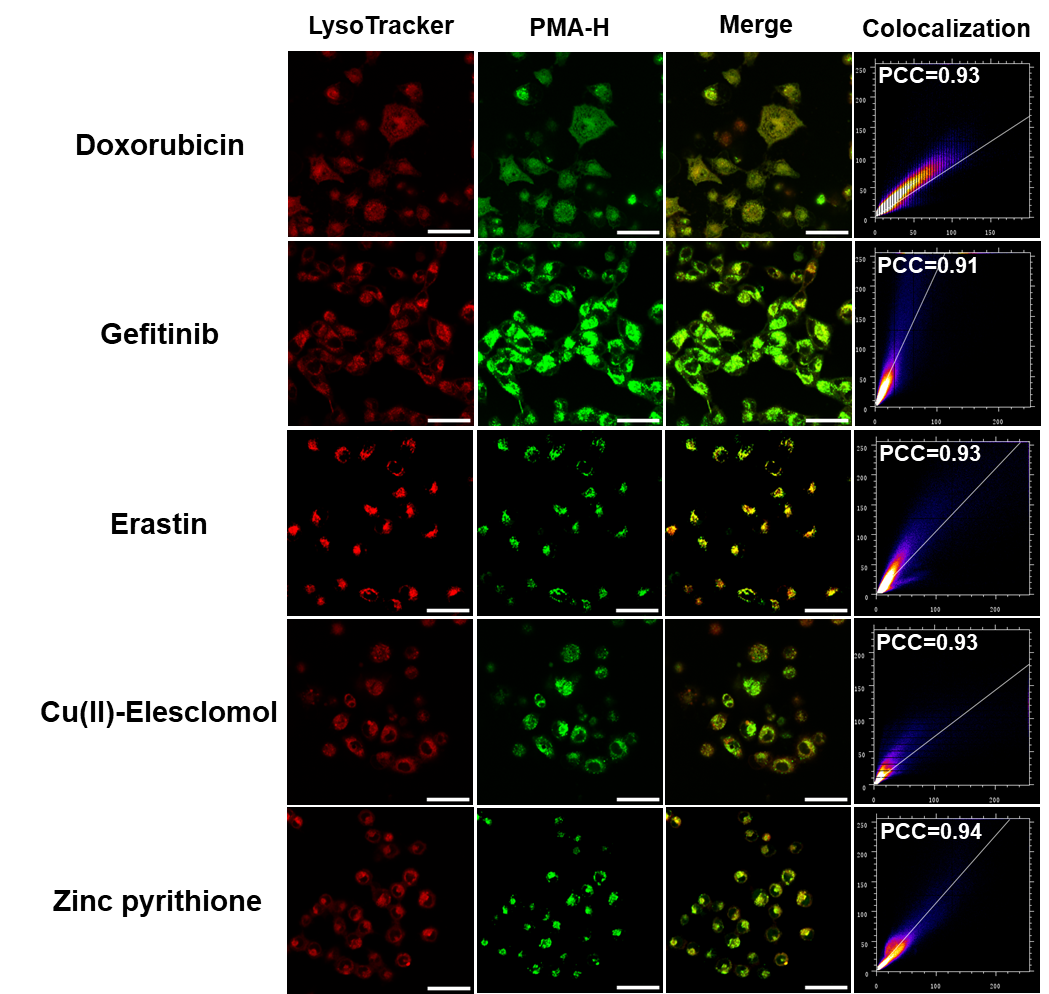


**Figure S13.** Colocalization of **PMA-H** and commercial lysosome tracker in drug-treated HeLa cells. HeLa cells were treated with **PMA-H** (5 μM) and LysoTracker™ Deep Red (0.1 μM) after incubation with Dox (5 μM), Gef (20 μM), erastin (20 μM), Cu(II)-elesclomol (0.05 μM), and ZnPT (1.0 μM). Emission from the red channel (LysoTracker), λ_ex_ = 638 nm, collected above 650 nm. Emission from the green channel (**PMA-H**), λ_ex_ = 488 nm, collected at 500–550 nm. (a_2_) Scatter plots for colocalization analysis between commercial dyes and **PMA-H**; Pearson's correlation coefficient (PCC) was calculated using ImageJ2. Scale bar: 50 μm.


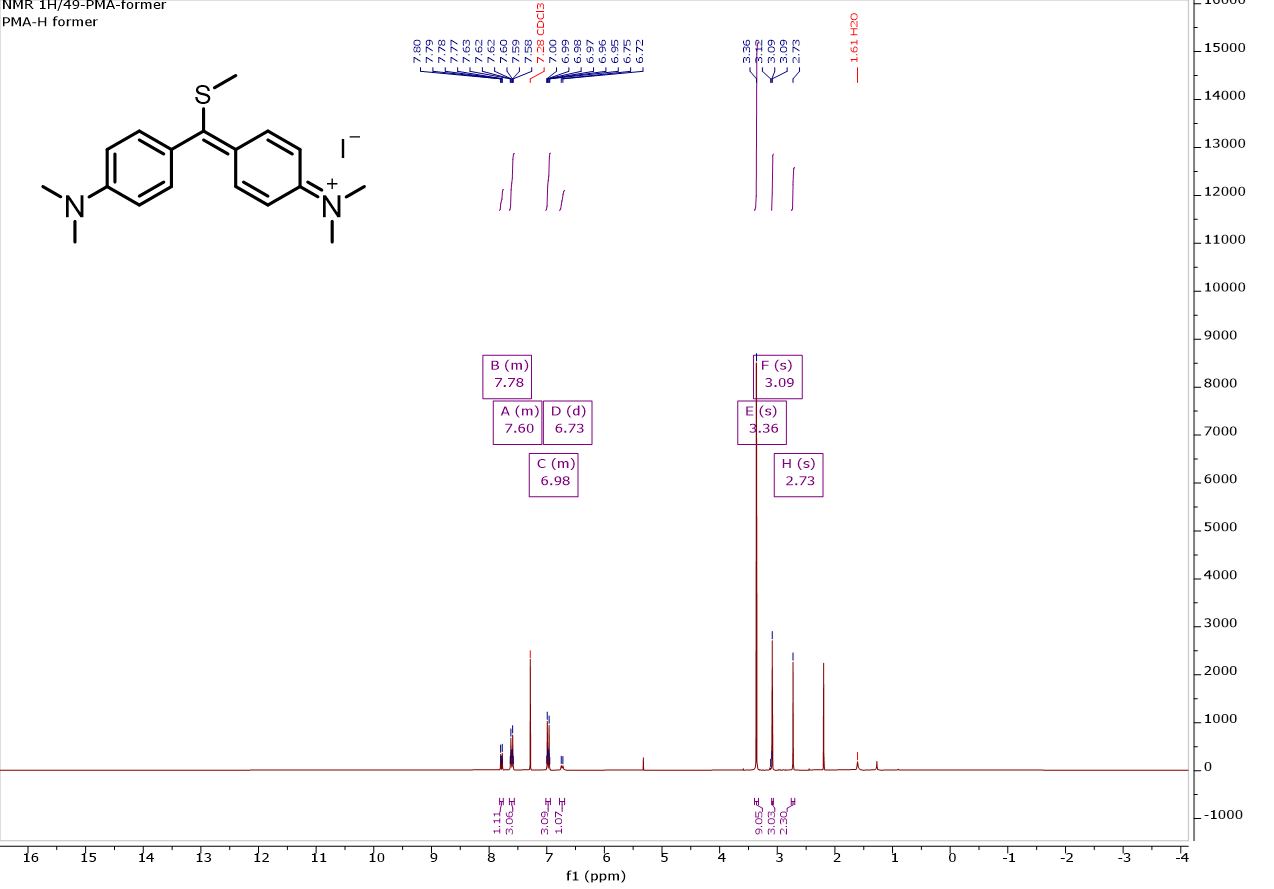


**Figure S14.** ^1^H NMR spectrum of compound **2** in CDCl_3_.


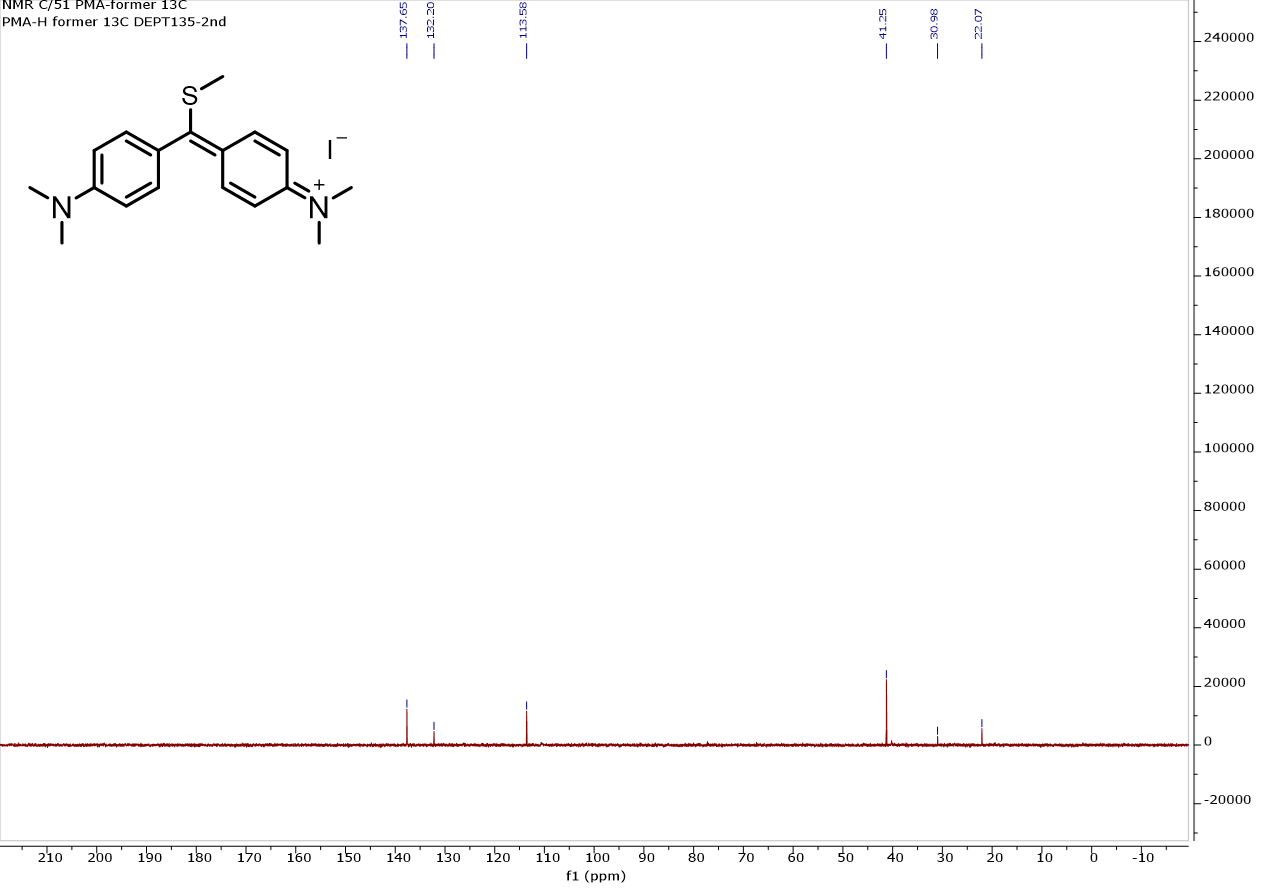


**Figure S15.** ^13^C NMR spectrum of compound **2** in CDCl_3_.


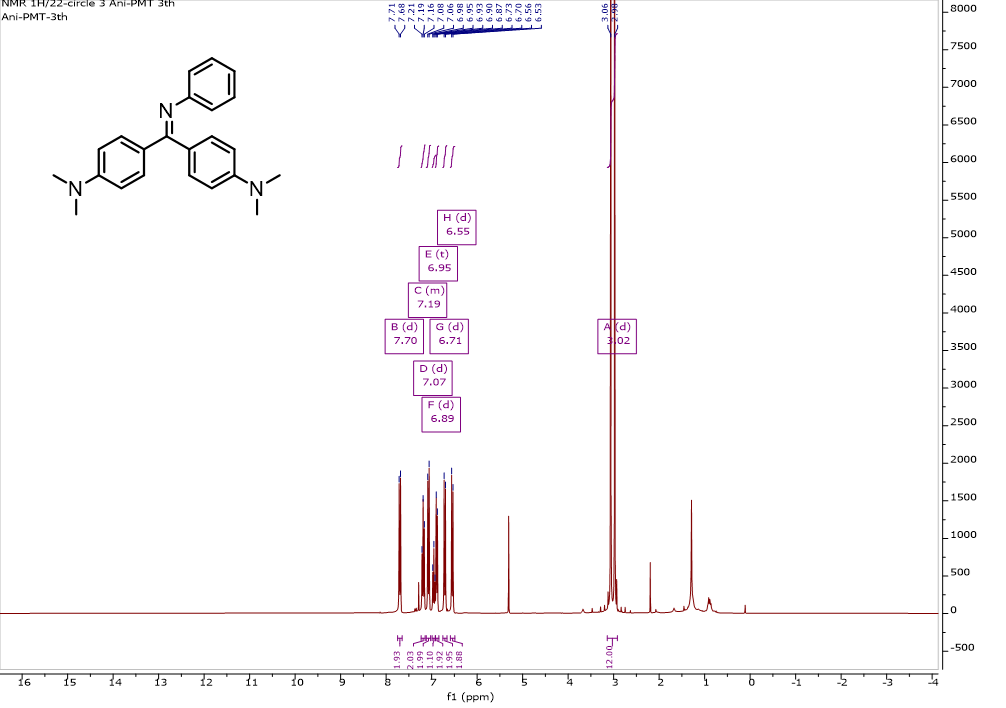


**Figure S16.** ^1^H NMR spectrum of **PMA-H** in CDCl_3_.

_
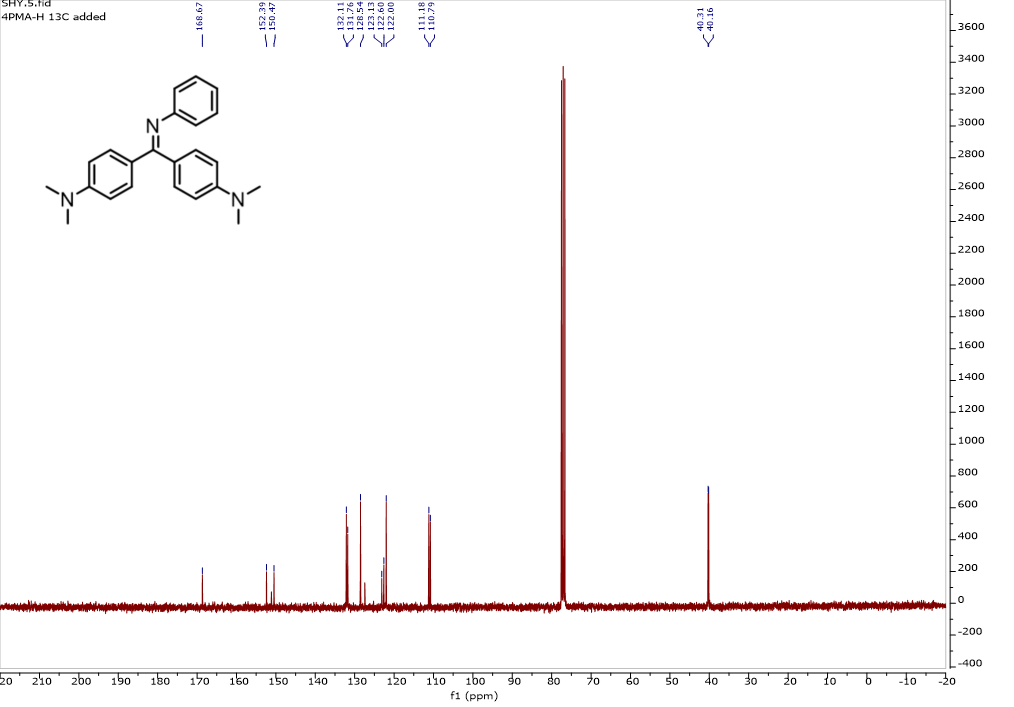
_

**Figure S17.** ^13^C NMR spectrum of **PMA-H** in CDCl_3_.


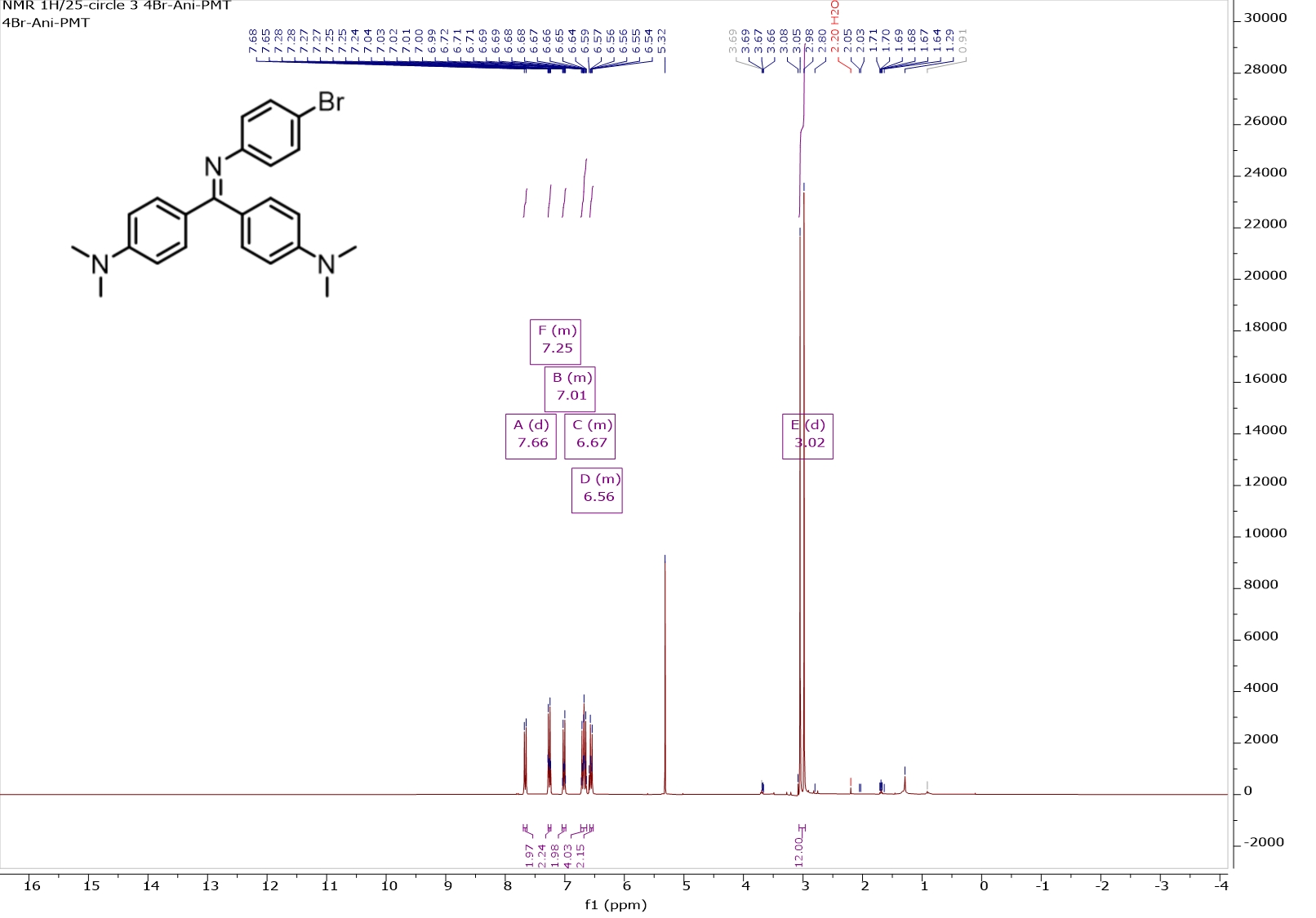


**Figure S18.** ^1^H NMR spectrum of **PMA-Br** in CDCl_3_.


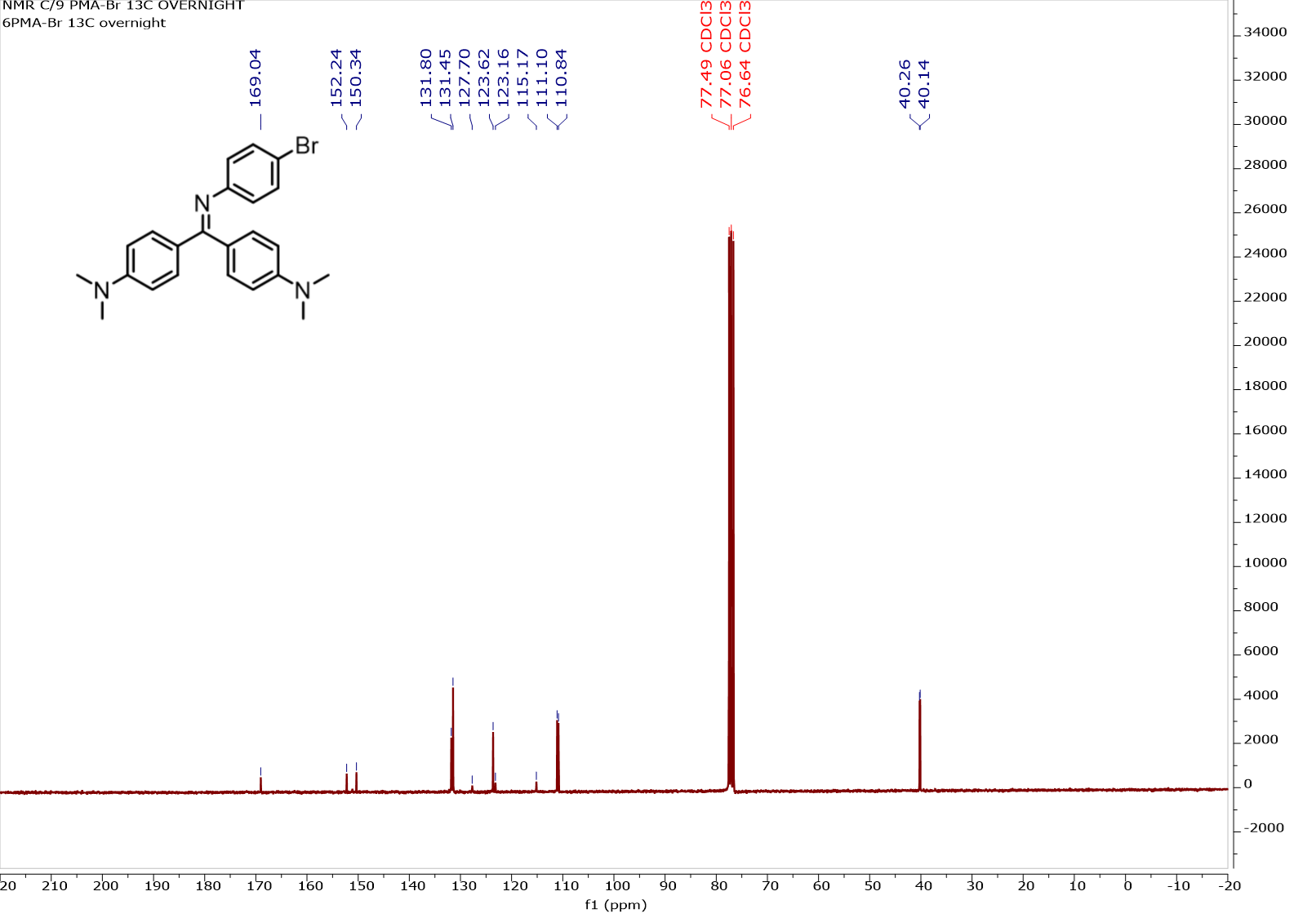


**Figure S19.** ^13^C NMR spectrum of **PMA-Br** in CDCl_3_.

**Figure S20.** ^1^H NMR spectrum of **PMA-I** in CDCl_3_.


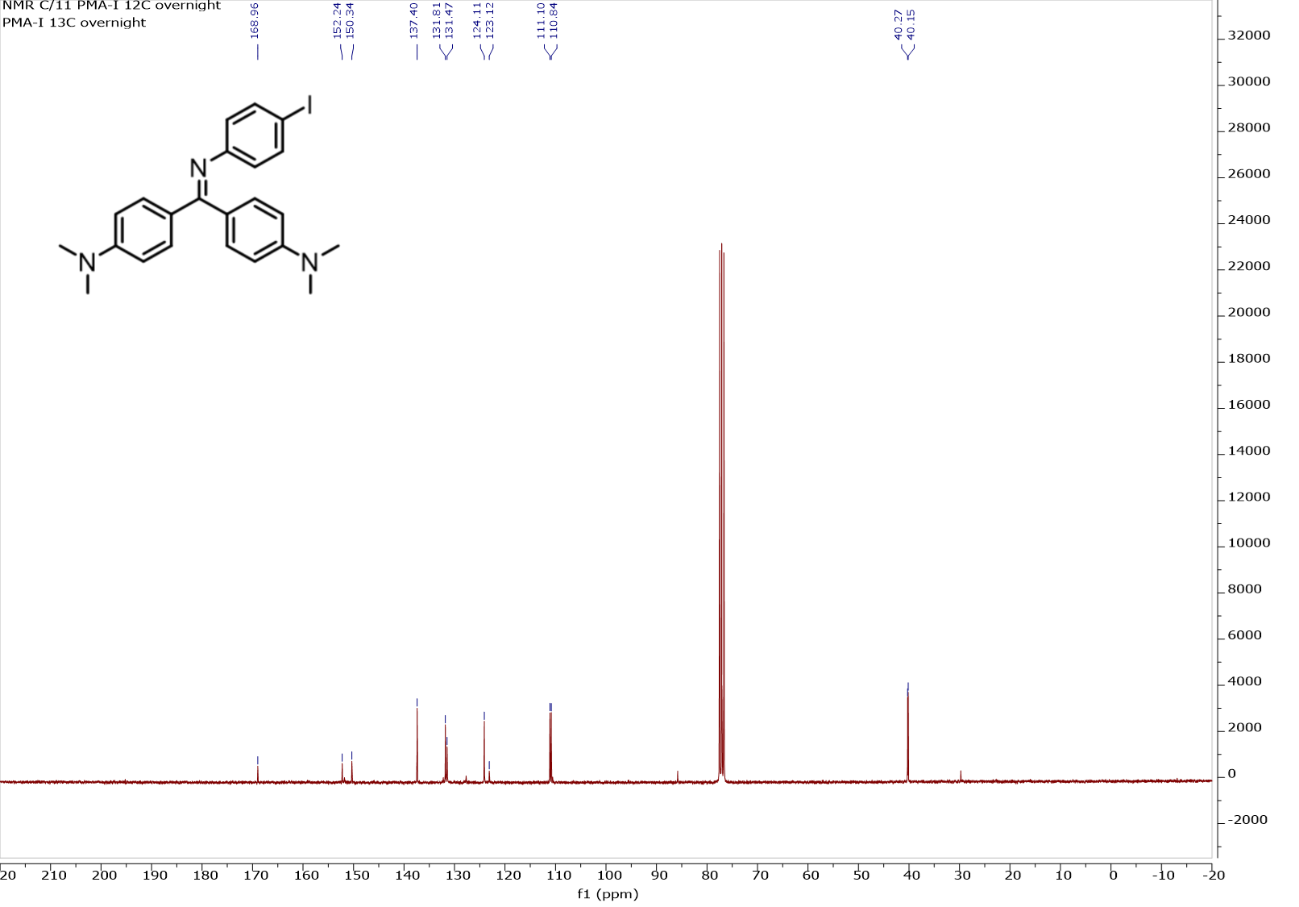


**Figure S21.** ^13^C NMR spectrum of **PMA-I** in CDCl_3_.

**Figure S22.** ESI-MS spectrum of **PMA-H.**

**Figure S23.** ESI-MS spectrum of **PMA-Br**.

**Figure S24.** ESI-MS spectrum of **PMA-I**.

**Reference**

[1] A. A. Yadav, D. Patel, X. Wu, B. B. Hasinoff, *J. Inorg. Biochem.* **2013**, *126*, 1-6.

[2] D. Magde, R. Wong, P. G. Seybold, *Photochem. Photobiol.* **2002**, *75*, 327-334.

[3] W. Chen, Z. Wang, M. Tian, G. Hong, Y. Wu, M. Sui, M. Chen, J. An, F. Song, X. Peng, *J. Am. Chem. Soc.* **2023**, *145*, 8130-8140.

[4] Y. Li, Z. Wu, Z. Huang, C. Yin, H. Tian, X. Ma, *Natl. Sci. Rev.* **2024**, *12*.

[5] L. Wang, Y. Xiao, W. Tian, L. Deng, *J. Am. Chem. Soc.* **2013**, *135*, 2903-2906.

[6] J. Liu, W. Zhang, C. Zhou, M. Li, X. Wang, W. Zhang, Z. Liu, L. Wu, T. D. James, P. Li, B. Tang, *J. Am. Chem. Soc.* **2022**, *144*, 13586-13599.

[7] J. Yin, M. Peng, W. Lin, *Anal. Chem.* **2019**, *91*, 8415-8421.

[8] S. Zhai, W. Hu, W. Wang, L. Chai, Q. An, C. Li, Z. Liu, *Biosens. Bioelectron.* **2022**, *213*, 114484.

[9] J. Tian, L. Zhang, Y. Qiao, J. Yang, R. Mengji, Y. Lee, S. Hong, Y. Duan, X. Zhou, J. S. Kim, J. Zhou, *Anal. Chem.* **2025**, *97*, 15244-15252.

[10] Q. Zan, L. Fan, L. Ma, Q. Yang, K. Zhao, Y. Huang, C. Dong, S. Shuang, *Sens. Actuators B: Chem.* **2023**, *397*, 134596.

[11] A. Tantipanjaporn, K. K.-Y. Kung, W.-C. Chan, J.-R. Deng, B. C.-B. Ko, M.-K. Wong, *Sens. Actuators B: Chem.* **2022**, *367*, 132003.
